# Growing palliative and end-of-life care research in the United Kingdom: Findings from a mixed methods survey

**DOI:** 10.1371/journal.pone.0357024

**Published:** 2026-09-15

**Authors:** Ikumi Okamoto, Alice Jin, Simon Noah Etkind, Donna Wakefield, Tracey McConnell, Anthony Byrne, Yesha Viren Dharamshi, Fliss Murtagh, Anne Finucane

**Affiliations:** 1 Clinical Psychology, School of Health in Social Science, University of Edinburgh, Scotland, United Kingdom; 2 Department of Psychology, School of Philosophy, Psychology, & Language Sciences, University of Edinburgh, Scotland, United Kingdom; 3 Primary Care Unit, Department of Public Health and Primary Care, University of Cambridge, Cambridge, United Kingdom; 4 Wolfson Palliative Care Research Centre, Hull York Medical School, University of Hull, Hull, United Kingdom; 5 School of Nursing and Midwifery, Queen’s University Belfast, Belfast, United Kingdom; 6 epartment of Palliative Medicine, Velindre University NHS Trust, Wales, United Kingdom; 7 Marie Curie Hospice Edinburgh, Scotland, United Kingdom; Bangladesh University of Health Sciences, BANGLADESH

## Abstract

**Background:**

Globally the number of people with an advanced progressive illness is increasing. Research is essential to inform policy, services and clinical practice to meet their needs. We investigated researcher perspectives on conducting palliative and end-of-life care research in the United Kingdom and what is needed to grow this field.

**Methods:**

We conducted a mixed-methods online survey of individuals conducting palliative and end-of-life care research in an academic institution or any care setting. Quantitative data were analysed descriptively, while qualitative data from open-ended questions were analysed using a Framework Method.

**Results:**

Overall, 256 people involved in palliative and end-of-life care research responded (80% female, 85% white, 83% aged over 35). Most worked at a university (79%), 67% worked full-time, 47% had a permanent contract and 9% were clinical academics. Many reported positive aspects of their working environment, such as a collaborative culture and research rigour. Nearly all (90%) wished to continue palliative and end-of-life care research during the next five years, however only 69% believed this was likely. Most common barriers were lack of funding (56%), heavy workload (42%), job insecurity (39%), and limited career progression opportunities (33%). To sustain and grow research activity, participants identified a need for: (i) Greater visibility of palliative and end-of-life care as a vital research field; (ii) Increased funding to support those undertaking palliative and end-of-life care research, across career stages and disciplines; (iii) Increased opportunities for collaboration and networking; (iv) Protected research time, and (v) Support to build a more diverse research community.

**Conclusions:**

Despite a committed workforce, palliative and end-of-life care research lacks visibility and funding. Increased visibility through knowledge exchange and impact activities, alongside more targeted research funding opportunities, are essential to build research capacity for the future.

## Introduction

Global patterns of population ageing and rising rates of non-communicable diseases are increasing the need for palliative care [1]. Worldwide, over 48 million people are projected to die with serious health-related suffering by 2060, representing an 87% increase from 2016 [1]. In the United Kingdom (UK), this trend is also evident and the need for palliative care is rapidly rising. [2,3],

The delivery of effective palliative care in ageing populations requires high-quality research to inform policy, service innovation and good clinical practice. However, analyses have consistently highlighted the dearth of funding for palliative care research compared with other disciplines [4]. A recent study of healthcare research funding in the UK revealed that out of 18,023 research awards in 2022, only 136 related to palliative and end-of-life care (PEOLC) research, representing only 0.26% of the £4.2 billion awarded [5]. The lack of funding constrains the extent to which researchers interested in palliative care can enter and remain in the field.

Challenges to developing the research workforce are evidence in all fields. In 2020, the Wellcome Trust commissioned a study of the general research workforce (n = 4,276) across the UK [6]. Their findings revealed that researchers are passionate about their work and value a collaborative, inclusive, supportive and creative working culture [6]. However, they experience significant challenges including institutional and funding systems that focus on quantity of outputs, narrow definitions of impact, publication pressure, job insecurity, and lack of managerial support. Experiences of discrimination and bullying were also reported [6]. In this study, over half of respondents were from the biomedical and biological sciences, and it is not possible to know the extent to which the findings apply to those conducting PEOLC research.

Existing UK-based studies on researcher perspectives regarding PEOLC research activity have been narrow in focus and limited to specific professional groups or regions. A 2004 mapping exercise in cancer research revealed a fragmented workforce and limited interdisciplinary collaboration [7]. A 2012 survey of cancer-related palliative care research leaders noted improvements in collaboration but highlighted persistent challenges. These included limited funding, lack of methodological expertise, lack of a clear research career structure, difficulties with research staff retention, and barriers for smaller groups aiming to build capacity and collaborate [8]. More recent studies have echoed these findings in specific researcher subgroups [9–11].

Findings from earlier studies on the perspectives of those conducting palliative care research are now outdated or narrow in scope; focusing on cancer research only [7,8], certain geographic regions [9], or specific professional groups (e.g., clinical academics) [11,12]. A more up-to-date and broader examination of the perspectives of those undertaking palliative care research is needed to guide growth, investment and capacity building.

### Aims

We sought to investigate perspectives on conducting PEOLC research in the UK; and to identify what is needed to sustain and grow PEOLC research to help address current and future health and social care challenges. To contextualise our findings, we also sought to compare perspectives of those conducting palliative care research with perspectives of the wider community of researchers in the UK, previously reported in the Wellcome Trust research cultures study [6].

## Methods

### Study design

We conducted a convergent mixed-methods study adopting the questionnaire variant of the convergent mixed methods research design [13,14]. We designed an online questionnaire consisting of closed and open-ended questions; whereby responses from the open-ended questions were used to triangulate and expand upon the quantitative findings. The qualitative data provided further details on participants’ experiences, priorities and suggestions that could not be captured through the closed-ended survey items alone. We report this study following the Checklist for Reporting Results of Internet E-Surveys (CHERRIES) [15].

### Research Advisory Group

We established a stakeholder Research Advisory Group to inform all aspects of the study. This group consisted of nine researchers, representing a mix of gender, age, ethnicity, research role and career stage (from PhD student to professor level). The group met on three occasions and provided feedback on research design, survey format and content, as well as preliminary findings.

### Questionnaire design

We developed an anonymous online questionnaire to address our research questions. We adapted some questions from the Wellcome Trust research culture survey [6,16] to allow comparison between those involved in palliative care research, with the broader research workforce in the UK. Our questionnaire contained 37 questions across seven webpages and took approximately 10–15 minutes to complete (S1 File). Data was collected online using Jisc Online Surveys (https://www.onlinesurveys.ac.uk/) between 26^th^ November 2024 and 16^th^ January 2025. Participants could opt-in to a prize draw and/or request a summary of the study results via a separate weblink (to maintain anonymity).

### Participants

We sought to recruit individuals involved in PEOLC research in the UK. We used the World Health Organisation (WHO) definition of palliative care as a starting point. According to the WHO, palliative care aims to improve the quality of life of patients facing challenges associated with life-threatening illness, as well as their families [17]. This includes addressing physical, psychological, social and spiritual concerns. Eligible participants included:

(i) those who describe themselves as palliative care researchers including any individuals who are currently or were recently engaged in research relating to PEOLC, advanced or life-limiting illness, death and dying, or bereavement.
(ⅱ) Those involved in palliative care research in any setting including university, hospital, hospice, or community.
(ⅲ) Those conducting palliative care research in any capacity including clinical academics conducting research as part of their role, and research nurses or academics at any stage in their research career from PhD student to professor level. Predoctoral researchers were excluded.

For brevity, we refer to all participants as ‘palliative care researchers’, based on their involvement in palliative care research. However, it is important to note that some participants may combine their palliative care research activity with other roles (e.g., clinical work) and/or other areas of research, and may not dedicate all their time to PEOLC research.

### Participant recruitment

We searched the websites of universities in the UK to identify PEOLC researchers, and emailed those with a publicly available email address with an invitation to our questionnaire (see S2 File) for search terms used to identify researchers involved in PEOLC via university websites**)**. We also advertised the survey at conferences, through relevant professional networks (e.g., Association for Palliative Medicine, Palliative Care Research Society, UK Palliative Trainee Research Collaborative, Scottish Partnership for Palliative Care) and via social media platforms (e.g., BlueSky and X). Potential participants accessed the participant information sheet, consent form and questionnaire using a link in the invitation email or social media advertisement. Once consent was provided, they continued to the questionnaire. No personally identifiable information was collected as part of the questionnaire.

### Data analysis

Quantitative data were analysed descriptively by the first author, IO, using SPSS v29. Sub-group comparisons were conducted to explore variations across key characteristics such as research roles, disciplines, and contract types. For comparison, we compared our findings with similar results representing the wider community of researchers in the UK, previously reported in the Wellcome Trust research cultures study [6]. Qualitative free-text responses were analysed in NVivo 14 using the Framework Method [18] by IO, AF, AJ, and YWD. Coding was both deductive and inductive. Data were first deductively coded based on four predetermined categories: (1) barriers to career progression, (2) support needed to remain research active, (3) strategies to promote PEOLC research to new researchers, (4) approaches to improve equity, diversity and inclusion. Within each of these categories, free-text responses were inductively coded, with researchers applying more detailed codes. The research team met weekly to compare codes across all categories and to develop a coding framework. Related codes addressing interconnected connected issues were re-grouped into five overarching themes. The five final themes identified therefore represent cross-cutting issues, drawing on both quantitative and qualitative data, rather than corresponding directly to the four predetermined categories.

### Data integration

Following collection of both quantitative and qualitative data concurrently, the research team analysed each dataset separately prior to consolidation and merging. During integration, the research team compared the findings from both datasets. This involved comparing common findings across each dataset and considering ways the results confirm, disconfirm and expand upon each other. The integrated results were presented via joint displays in PowerPoint, and then shared, discussed and refined during full team meetings. The qualitative findings did not modify the quantitative results themselves; rather, they deepened and expanded their interpretation. The integration of both datasets allowed development of results and interpretations that expanded our understanding of participant perspectives on conducting PEOLC research in the UK, with qualitative findings contextualising and adding detail to the quantitative findings.

### Ethical approval

The University of Edinburgh’s Health in Social Science ethics committee granted ethical approval on 21^st^ November, 2024 (Case ID: 24–25CLPS007). Participants were asked to read an online participant information sheet and to complete an online consent before progressing to the questionnaire. No data that could identify a participant’s information was requested.

## Results

Overall, 256 participants completed the questionnaire. All quantitative questions were compulsory, whereas the six open-ended questions were optional. Of the 256 participants, 244 responded to at least one open-ended question, yielding 966 qualitative responses altogether (excluding non-substantive responses such as “unsure,” “N/A”).

### Participant characteristics

Most were white (85%), female (80%), and aged 35 or over (83%). Most worked at a university (79%), 67% worked full-time, and 47% had a permanent contract. Most were paid to conduct research as part of their role, while a minority (12%, n = 31) conducted research in their own time (e.g., honorary research staff). Time spent on research activities ranged from less than 5 hours to 50 hours per week. The full sample characteristics are presented in S1 Table.

**Fig 1:**
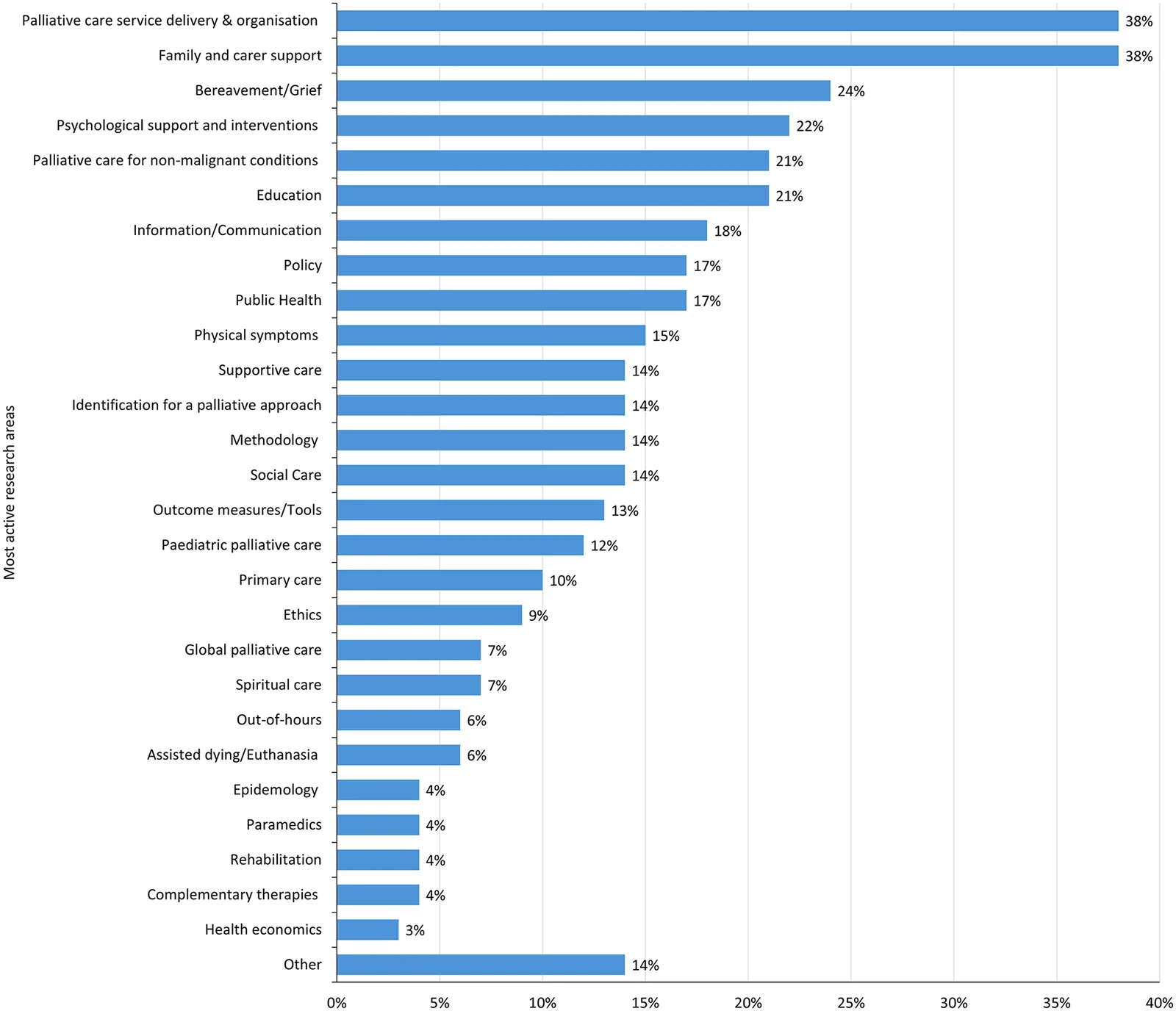
Most commonly reported research areas *Participants were asked to select up to five research areas.

When compared with the Wellcome Trust’s UK-wide research culture survey [6,19], our sample had a higher proportion of females (80% vs 60%), and were older with only 17% under the age of 35 (vs 39%). However, a large proportion of our participants were early on in their research career, with 37% having worked in PEOLC research for less than five years. ‘Palliative care service delivery and organisation’ and ‘family and carer support’ were most often reported as an area in which respondents were research active (Fig 1).

### Perceived likelihood of remaining research active

Participants were positive about many aspects of their work environment (Table 1), with most agreeing that rigour is valued by their institution (81%), that they work in a collaborative culture (78%), that creativity is welcomed (69%), and that they feel listened to if they raise a concern (66%). Overall, participants conducting palliative care research were more positive about all aspects of their working environment compared to the general research workforce as indicated by the Wellcome Trust Survey. (Table 1).

**Table 1:** Comparison of rates of participant agreement with statements relating to their current working environment: Palliative care researchers versus researchers in general.

How far do you agree or disagree with the following statements relating to your current working environment?	Palliative care survey respondents who agree ^a^, % (N=256)	Wellcome Trust survey respondents who agree ^b^,% (N=4065)
1. Rigour of results is considered an important research outcome by my institution/workplace	81%	69%
2. My working environment promotes a collaborative culture	78%	61%
3. Creativity is welcomed within my working environment in all its forms	69%	60%
4. I am confident that my institution/workplace would listen and take action if I raised a concern	66%	47%
5. The culture around research in my working environment supports my ability to do good quality research	62%	57%
6. My institution/workplace provides me with support to navigate the grant application process	59%	48%
7. My working environment promotes a good work-life balance	57%	47%
8. My institution/workplace’s expectations of me to undertake a number of roles leaves me little time for research	39%	44%
9. My working environment hinders researchers getting on with their research	35%	39%
10. Healthy competition is encouraged within my working environment	35%	32%
11. My institution/workplace could do more to ensure research practices do not cut corners	24%	46%
12. Unhealthy competition is present within my working environment	18%	42%
13. My institution/workplace places more value on meeting metrics, than it does on research quality	16%	43%
14. My institution/workplace values speed of results over quality	12%	32%

Notes: ^a^We assessed agreement using a 5-point scale: 1 = strongly disagree, 2 = disagree, 3 = neither disagree nor agree. 4 = agree, 5 = strongly agree. Agree includes 4–5. ^b^The Wellcome Trust survey used a 7 point scale: 1 = Strongly disagree, 4 = Neither disagree nor agree, 7 = Strongly agree; Agree includes 5–7 [19].

**Fig 2:**
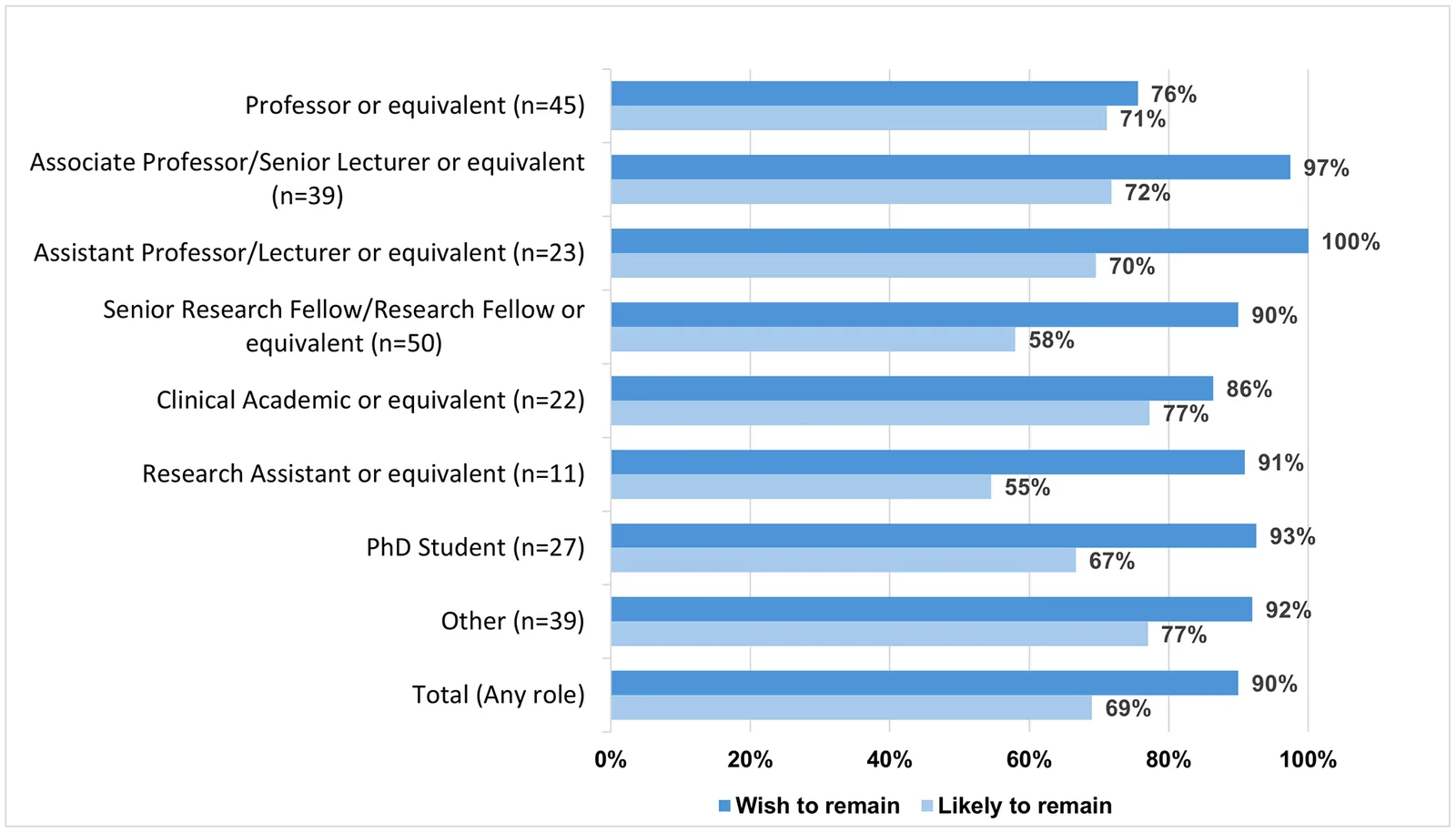
Overall agreement with “wish to remain” and “likely to remain” in PEOLC research over the next 5 years (N = 256).

**Fig 3:**
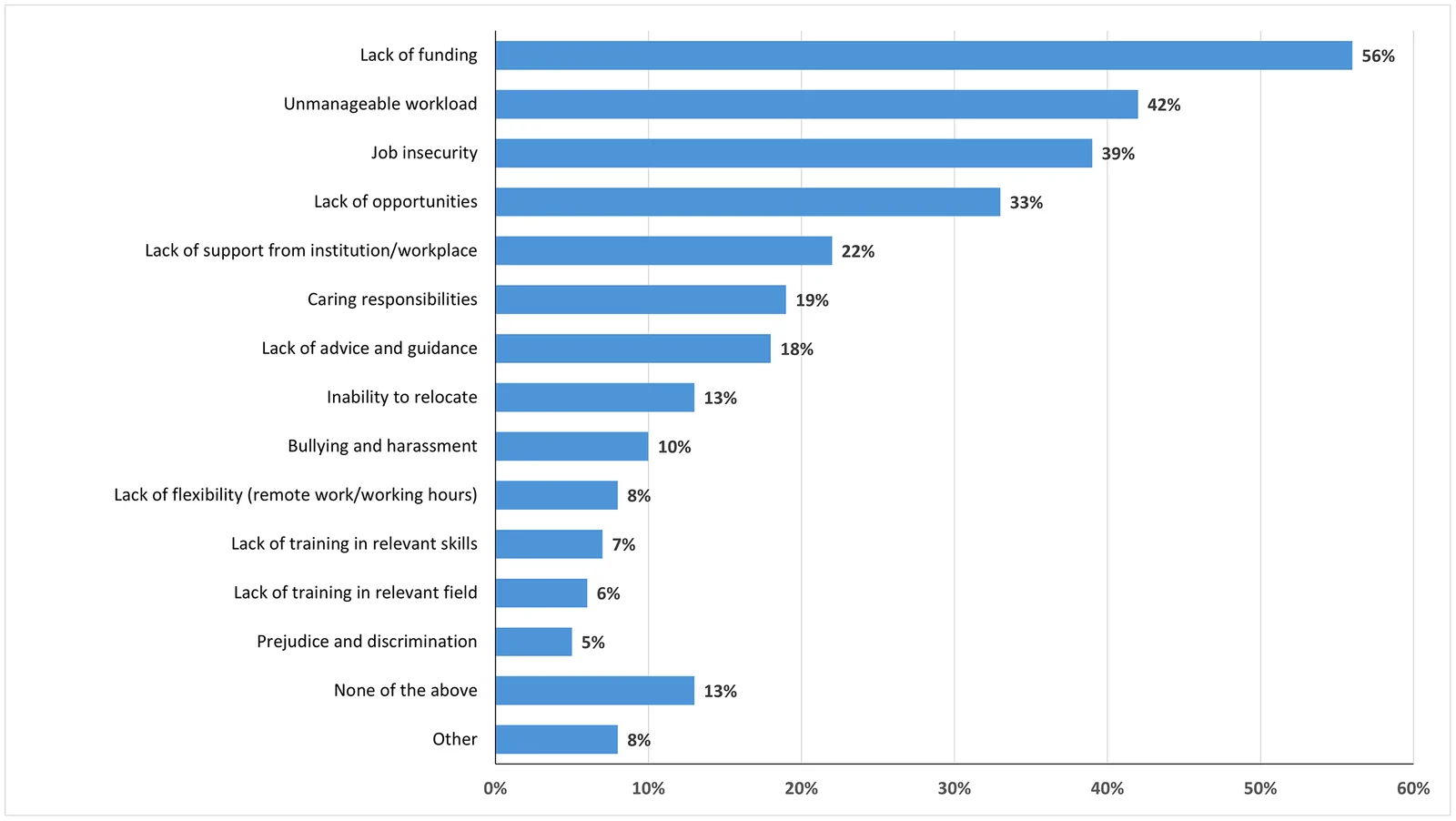
Barriers to a successful career in PEOLC research *Participants were asked to select all that apply.

Despite these favourable perceptions, only 69% believed they were likely to continue working in PEOLC research in the next five years, although the majority (90%) expressed a wish to do so. The gap was greatest for research fellows and research assistants (Fig 2). By contract type, most researchers on permanent and fixed-term contracts wished to remain in the field (90% and 89%, respectively); however, only 75% and 60% expected to still be active in this area in five years. The most frequently reported barrier to a successful career in palliative care research was lack of funding, followed by unmanageable workload, job insecurity, and lack of opportunities (Fig 3).

### Sustaining and growing PEOLC research

**Fig 4:**
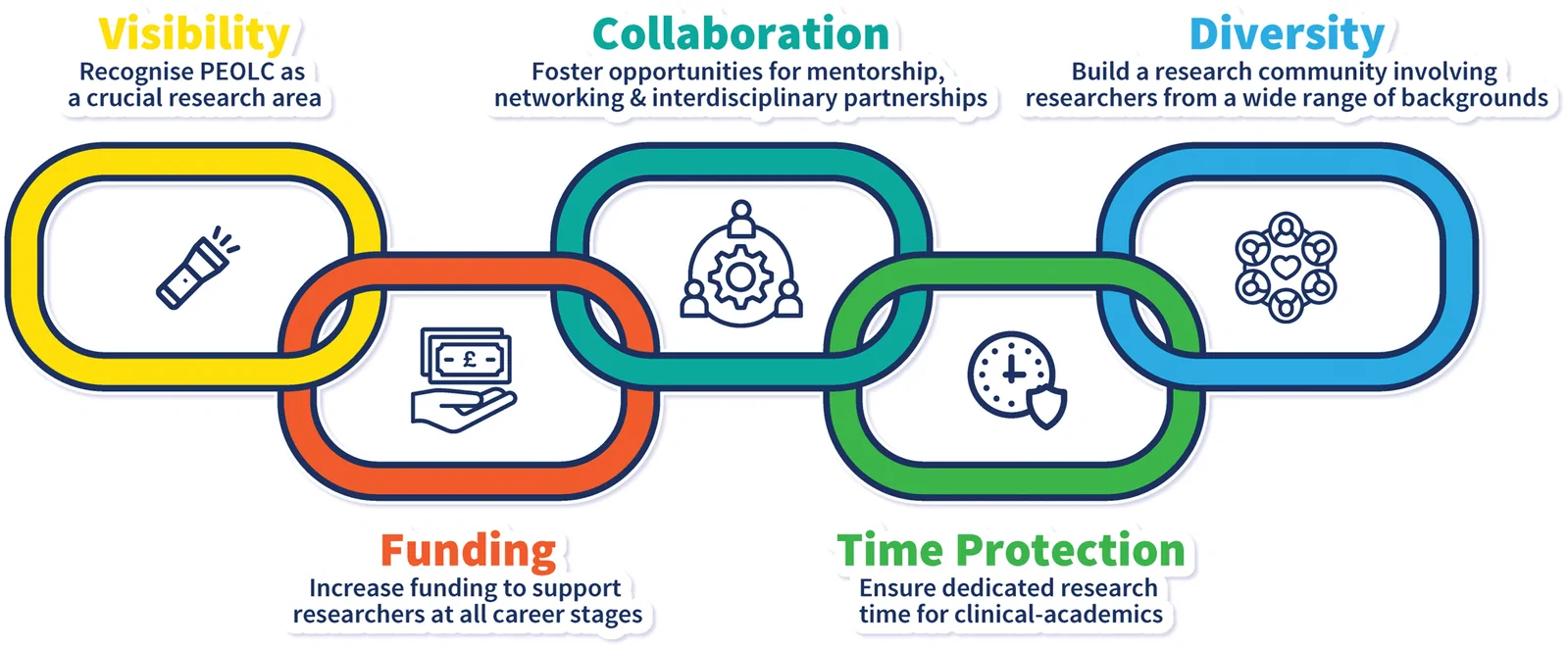
Factors influencing the palliative care research workforce development and sustainability*. *Note: Fig 4 was created by the corresponding author (AF) using Napkin AI, an artificial intelligence tool designed to transform text descriptions into diagrams.

Drawing on the quantitative and qualitative data, we generated five themes describing participant perspectives on sustaining and growing PEOLC research. These were: (i) visibility of PEOLC as a vital research field (ii) research funding across career stages and disciplines (iii) opportunities for collaboration, networking and mentorship; (iv) protected research time and (v) support to build a more diverse research community (Fig 4). We elaborate on each theme in the following sections.

#### Visibility of PEOLC research.

Participants perceived an underinvestment in PEOLC research compared with curative, disease‑specific research, limiting the development of the field.

*“Palliative care and end-of-life care are often not as well funded as other disease areas (such as cancer, heart disease, etc.). Much research funding is focused on curative research and treatment innovations, while palliative care research is often neglected.” (ID19, Clinical Academic, Full-time, Fixed-term, Medicine)*

The holistic nature of palliative care, which involves addressing physical, psychological, social and spiritual concerns of the terminally ill person and their family, means that the research area is broad, and requires expert input from a wide range of fields. Raising awareness of the breadth of the field, in order to connect with other experts, was viewed as important to grow PEOLC research.

*“I think we need to be clearer about the broad scope of palliative care, and the opportunities where we need help and need experts from diverse fields, so people can get excited about what they can bring to palliative care from elsewhere - from psychology to engineering to the arts. This might include presenting our work at conferences beyond palliative care, and forming new collaborations, where people can see how their existing work might also apply to people with life-limiting illness / advanced disease.” (ID163, Assistant professor/Lecturer or equivalent, Full-time, Permanent, Psychology)*

Participants identified a need to improve understanding of PEOLC research, including correcting misconceptions about its scope. Some argued for wider dissemination of findings beyond palliative care audiences, to increase visibility, and reduce its separateness from other disciplines and specialities.

*“Greater visibility; other clinicians do not know what palliative care research entails, usually due to an incorrect understanding of the scope of palliative care. Greater dissemination of research both among the public and academics in other fields.” (ID236, Clinical Academic, Full-time, Fixed term, Medicine)*

Participants also described a need to highlight the attractiveness of the field, and reduce concerns that it may be much more emotionally demanding that other research areas.

*“Reassurance that [PEOLC] research is not scary/more emotionally demanding than other fields - this was my concern when I started my first role.” (ID2, Senior Research Fellow/Research Fellow, Full-time, Fixed-term, Social Science)*

Participants highlighted a need for researchers to get better at demonstrating the relevance of their findings for policy, health and social care in order to increase visibility and attract more funding.

*“Evidence that what we do works and impacts positively on health and social care, so that palliative care is funded by the government. (ID186, Assistant Professor/Lecturer, Full-time, Fixed-term, Medicine)*

#### Research funding across career stages and disciplines.

**Fig 5:**
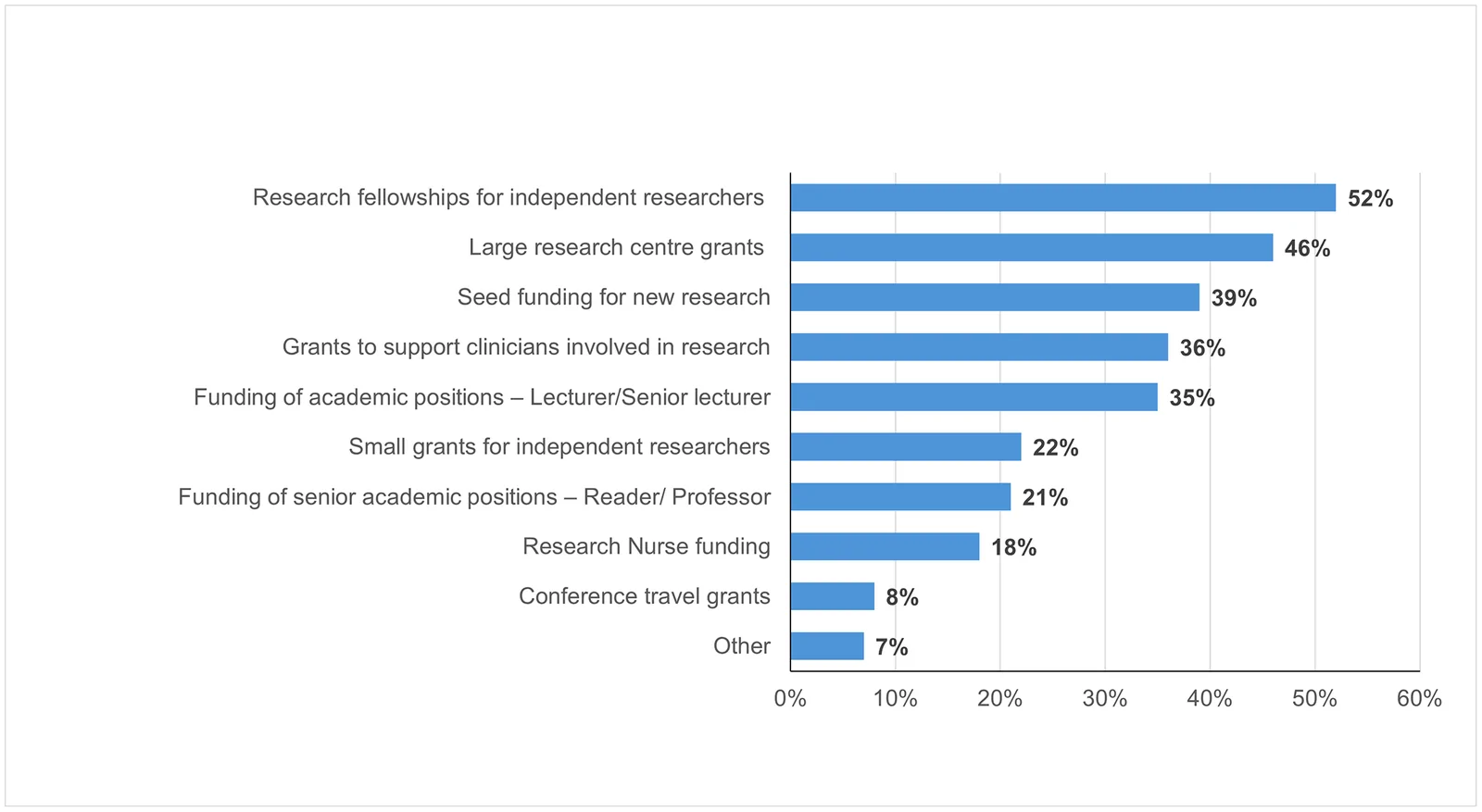
Most needed funding to ensure the growth of palliative and end-of-life care research activity *Participants could select up to three categories.

A lack of funding was reported as a research barrier by 56% of participants (Fig 3), and was a common concern for researchers, irrespective of level of seniority and experience (S1 Fig). A wide range of funding types were reported as needed to grow the field. (Fig 5).

Research fellowships for independent researchers were most frequently selected as the ‘most needed’ type of funding to grow research activity (Fig 5). These were viewed as especially important for early and mid-career researchers with many reporting a need for ‘*longer term contracts and opportunities for fellowships*” (*ID61, Senior Research Fellow, Part-time, Fixed-term, Nursing)*. Participants also noted the desire for careers advice, and support to write fellowship and grant applications.

*“Career guidance and advice about what steps to take next. Support to write grant/fellowship applications. (ID1, Clinical academic, Full-time, Fixed-term, Medicine)*

Job insecurity was considered a barrier to a successful career by 39% of all participants (Fig 3), and by over two-thirds of those employed on a fixed-term contract. Consequently, funding to support researchers to move from a fixed-term to permanent contract was identified as important in growing PEOLC research and building research workforce capacity. Research teams are often composed of fixed-term contract researchers whose continued employment relies on securing external funding. This creates uncertainty and diverts attention away from current work as efforts are expended on horizon scanning for future funding and continuity planning.

*“More opportunities for permanent positions then I can concentrate on doing actual research, rather than worrying about whether myself and my team will have a job in a couple of years”. (ID199, Associate professor/Senior Lecturer, Full-time, Fixed-term, Health Sciences)*

Employment precarity was described as affecting not only individual security but also the sustainability of PEOLC research projects and teams. The reliance on fixed-term contracts was seen as a systemic inefficiency, disrupting project progress and staff career development.

*“Reliance on fixed-term contracts for project researchers, making it difficult to run projects to time and maintain staff continuity and support career development.” (ID176, Senior Research Fellow/Research Fellow, Part-time, Fixed-term, Medicine)*

Participants emphasised the need for permanent or longer-term contracts to pursue longer-term, more ambitious research projects.

*“Better job security which would motivate people to plan more long-term projects and grant applications.” (ID174, Senior Research Fellow/Research Fellow, Full-time, Fixed-term, Allied Health Profession)*

Larger research centre investment was seen as key to retaining skilled researchers and building research infrastructure (Fig 5). Participants stressed that without such funding, the field remains overly dependent on short-term, individual grants, which are uncertain and insufficient to create the environment necessary for cultivating future talent in the research workforce.

*“Funding to develop a centre at our university to build up the capacity; currently it relies on a specific few people and without a consistent funding stream it is difficult to retain postdoctoral researchers.” (ID6, Professor or equivalent, Full-time, Permanent, Social Science)*

Given the prevalence of fixed-term contracts, bridging funding (i.e., funding to support researchers between contracts) was viewed as essential in sustaining activity and supporting career progression, especially for postdoctoral researchers. Clinical academics also reported the need for bridging funding to allow them to develop future projects and funding applications, as they have no time to plan applications once they return to their clinical role.

*“Bridging funding to allow researchers to maintain funding between grants. Without this, promising researchers are likely to drop out of academia due to lack of job security.” (ID227, Senior Research Fellow/Research Fellow, Part-time, Fixed-term, Social Science).*

Rigid training structures and lack of flexibility in clinical academic pathways were also described as creating barriers to research. Funding to support more flexible pathways were needed to enable clinicians to combine research with clinical work and grow the field.

*“More options to work as a researcher and a clinician than a fixed training programme that has a long-term commitment. Flexibility to apply for grants for 1 - 2 years research alongside clinical training. Encouragement from the medical training programme to explore other opportunities rather than just work full time clinically.” (ID84, Senior Research Fellow/Research Fellow, Part-time, Other contract type, Medicine)*

Grant funding to support the very early or late stages of a project were viewed as needed. Initial seed funding was vital to support collaboration, patient and public involvement and time to develop a research idea. Towards the end of a project, impact funding was essential to support wider dissemination, engagement, and impact activities.

*“A lot of our research has risen from seed corn funding, and this is vital.” (ID95, Professor or equivalent, Part-time, Permanent, Nursing)*

*"Follow-on funding for grants/projects (even if project was initially unfunded) to enable dissemination and impact" (ID6, Professor or equivalent, Full-time, Permanent, Social Science)*

Researchers working in the ‘social sciences, education and humanities’ most frequently identified ‘lack of funding’ as a barrier to conducting research (S2 Fig). Social scientists highlighted the multidisciplinary nature of the field and called for more funding schemes for critical approaches using sociological theory, alongside approaches that are biomedical and positivistic.

*“Grant funding that recognises and prioritises interdisciplinary and multidisciplinary projects mixing clinical contexts with social sciences, regulatory issues and legal frameworks.” (ID73, Professor or equivalent, Full-time, Permanent, Other discipline)*

#### Collaboration and networking.

**Fig 6:**
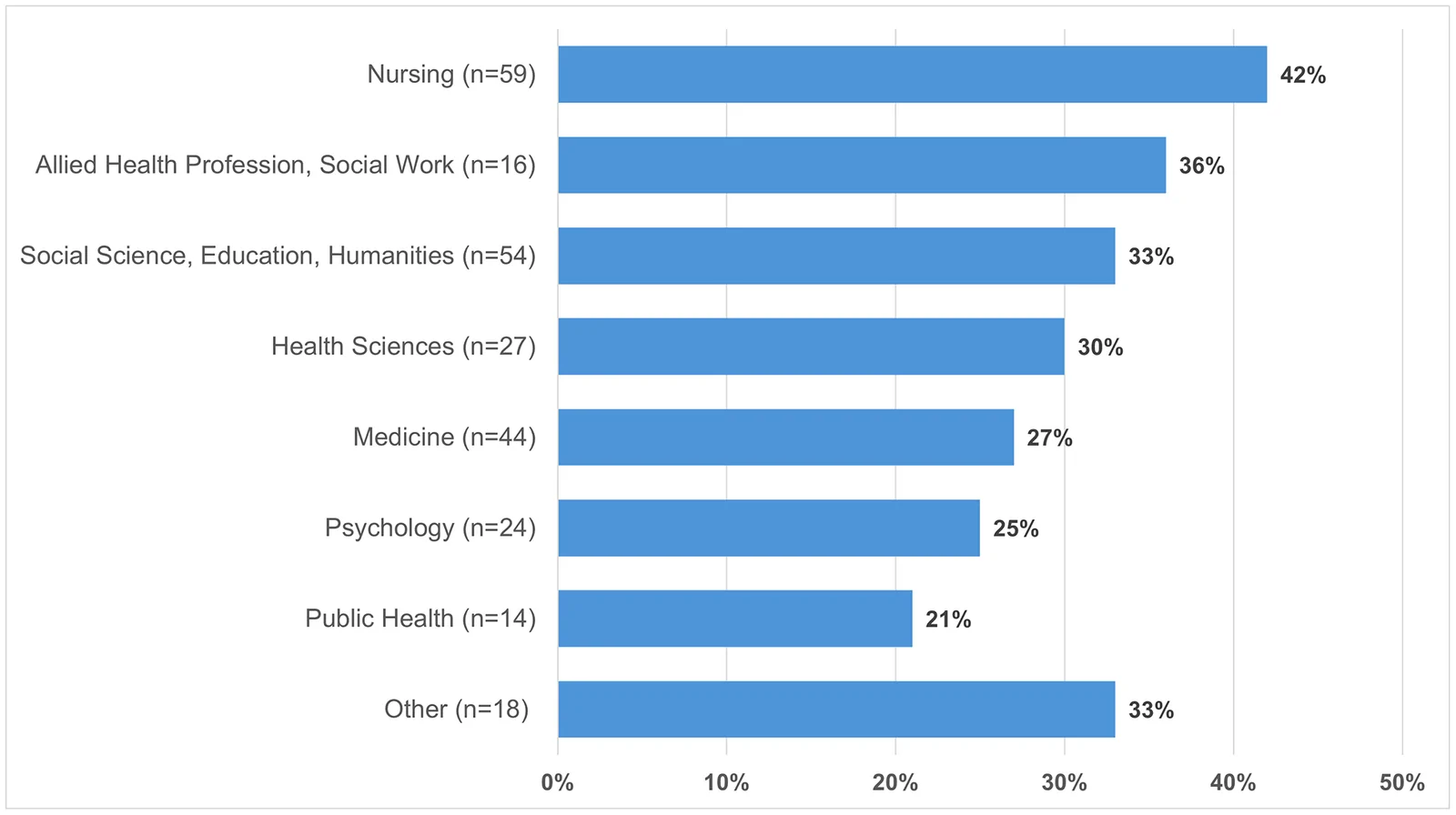
Proportion of participants reporting “lack of opportunities” as a barrier to a successful career in palliative and end-of-life care research, by discipline.

Lack of opportunities was identified as a significant barrier to a successful career by one-third of participants (Fig 3), and was most frequently identified as a barrier by those in nursing (Fig 6). Networking and mentorship opportunities were considered key enablers of research activity and career development, creating opportunities for researchers to connect and plan future work together, but these were often lacking.

*“Career mentorship opportunities to help with developing [a] research profile, improve efficiency of activities, and grant application strategies. Opportunities to meet with potential future collaborators beyond conferences, e.g., networking event, grant development workshops, invitations to seminars etc” (ID125, Assistant Professor/Lecturer, Part-time, Permanent, Social Science)*

Researchers working independently at an academic institution, who were not part of palliative care group or team, reported feeling isolated. Working alone meant that opportunities for development were very limited. They could see the value in research centres where people worked in larger palliative care research groups.

*“Specialist research centres where researchers can work alongside other people in the field, I am the only one within my university actively working in this area which can sometimes be isolating.” (ID134, PhD student, Social Science)*

Teams where there was a critical mass of PEOLC researchers were particularly attractive in terms of opportunities for mentorship, learning and career progression. Larger groups also provided access to strategic leadership, and greater opportunities for collaborating on future projects.

*“Useful to have research centres/strategic leadership that can provide some continuity - although these need to act as a hub for researchers working in wider settings - so no one is 'orphaned'.” (ID164, Senior Research Fellow/Research Fellow, Part-time, Fixed-term, Health Sciences)*

Joining established research groups was considered key for networking opportunities, knowledge exchange and skills development, particularly for early-career researchers. Networks need not be institution-based, but just needed to provide peer and practical support, as well as a space for information sharing.

*“Opportunities to collaborate with others and join research teams—early career researchers can learn much from this.” (ID30, Senior Research Fellow/Research Fellow, Full-time, Fixed term, Psychology).*

Mentorship was viewed as helpful in supporting career progression. A lack of mentorship hindered skills development and research progress for some participants.

*“I suffered from not having a mentor or perhaps a champion, someone who could nurture my career and help me navigate the pitfalls of research (lack of investment, lack of strategic direction and often poor leadership)”. (ID 172, Assistant Professor/Lecturer, Part-time, Permanent, Nursing)*

#### Protected time.

**Fig 7:**
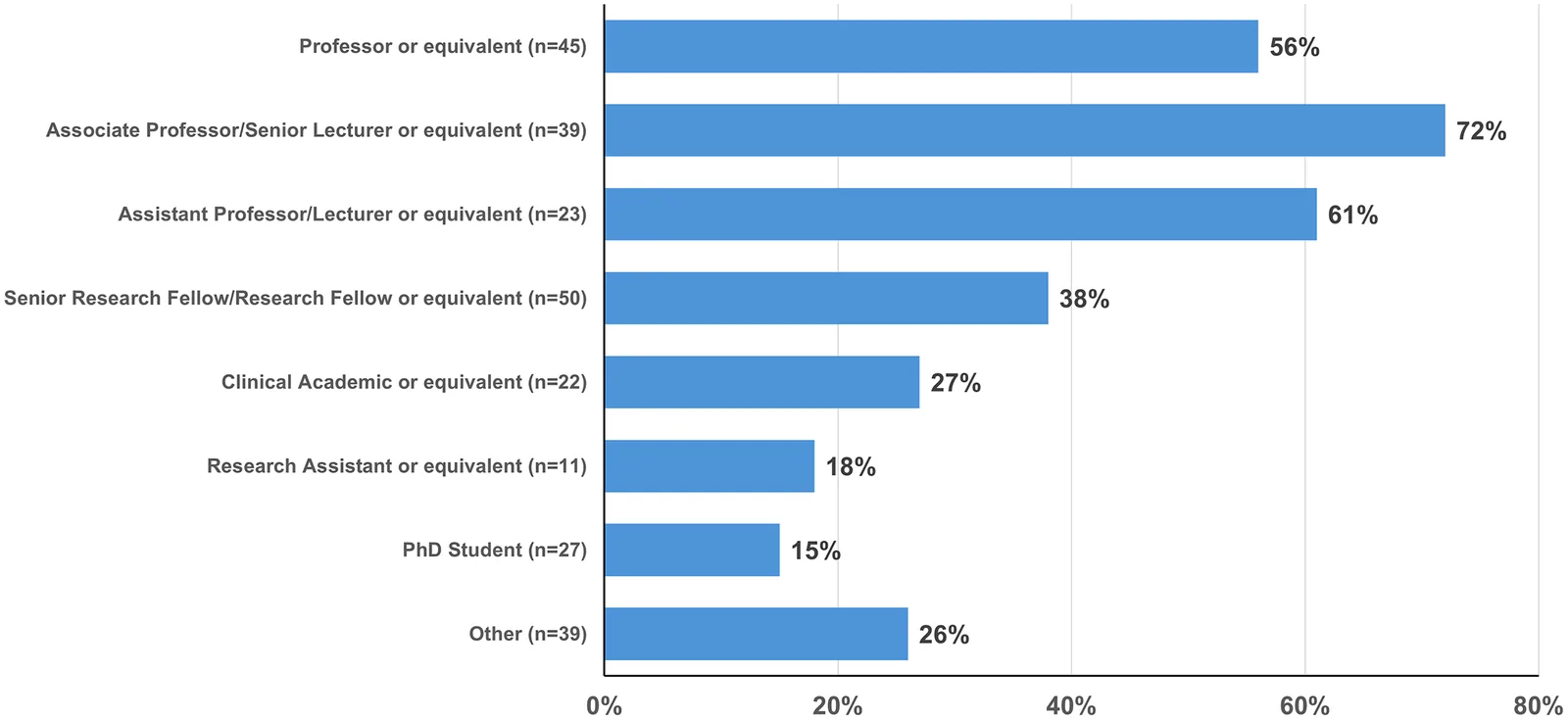
Proportion of participants reporting “unmanageable workload” as a barrier to a successful career in palliative or end-of-life care research, by professional role.

Unmanageable workload was reported as a barrier to conducting research by 42% of participants (Fig 3), and was particularly pronounced among those at the associate professor and senior lecturer level (Fig 7). Allocating time for research, given teaching, clinical, and administrative duties was a major challenge.

*“Institutional pressures that mean that educational pressures take precedence over research, particularly the ability to carve out time to be creative and consider future grant applications.” (ID144, Associate Professor/Senior Lecturer, Full-time, Permanent, Nursing)*

Unmanageable workload in the higher education sector resulted in little time for research grant planning, and could lead some to leave the sector and return to a clinical role. This was especially the case for those juggling multiple roles.

*“I am doing a small research project within my clinical role. I moved out of academia as I found the academic environment very difficult as a contract researcher and a part-time lecturer. The lecturing took up so much time it left me no time or head space to apply for grant funding.” (ID7, Former research fellow, current clinical academic, Part-time, Nursing)*

For those conducting research in clinical settings, clinical responsibilities were described as limiting their research capacity to lead research projects. As a result, opportunities to apply for grants, and lead studies were curtailed.

*“Clinical workload, so lack capacity to lead research projects.” (ID191, Other role, Full-time, other contract type, Medicine)*

To sustain engagement in research for those working in clinical settings, protected time was deemed essential. Clinical academic posts were viewed as particularly valuable in ensuring protected research time and future career progression.

*“Jointly funded clinical academic posts …. so that research time is protected to deliver research and build capacity to support career progression and succession planning.” (ID46, PhD Student, Part-time, Fixed-term, Nursing)*

Research governance requirements for PEOLC research were viewed as complex and time-consuming to navigate, especially for early career researchers. Some participants described a need for support to gain an understanding of ethics and governance, which also needed to be factored into research plans.

*“Support with ethics and governance applications; often [PEOLC] research is viewed as 'sensitive' and this can make it difficult for new researchers to navigate ethics and governance procedures.” (ID6, Professor or equivalent, Full-time, Permanent, Social Science)*

#### Diversity and inclusion.

**Fig 8:**
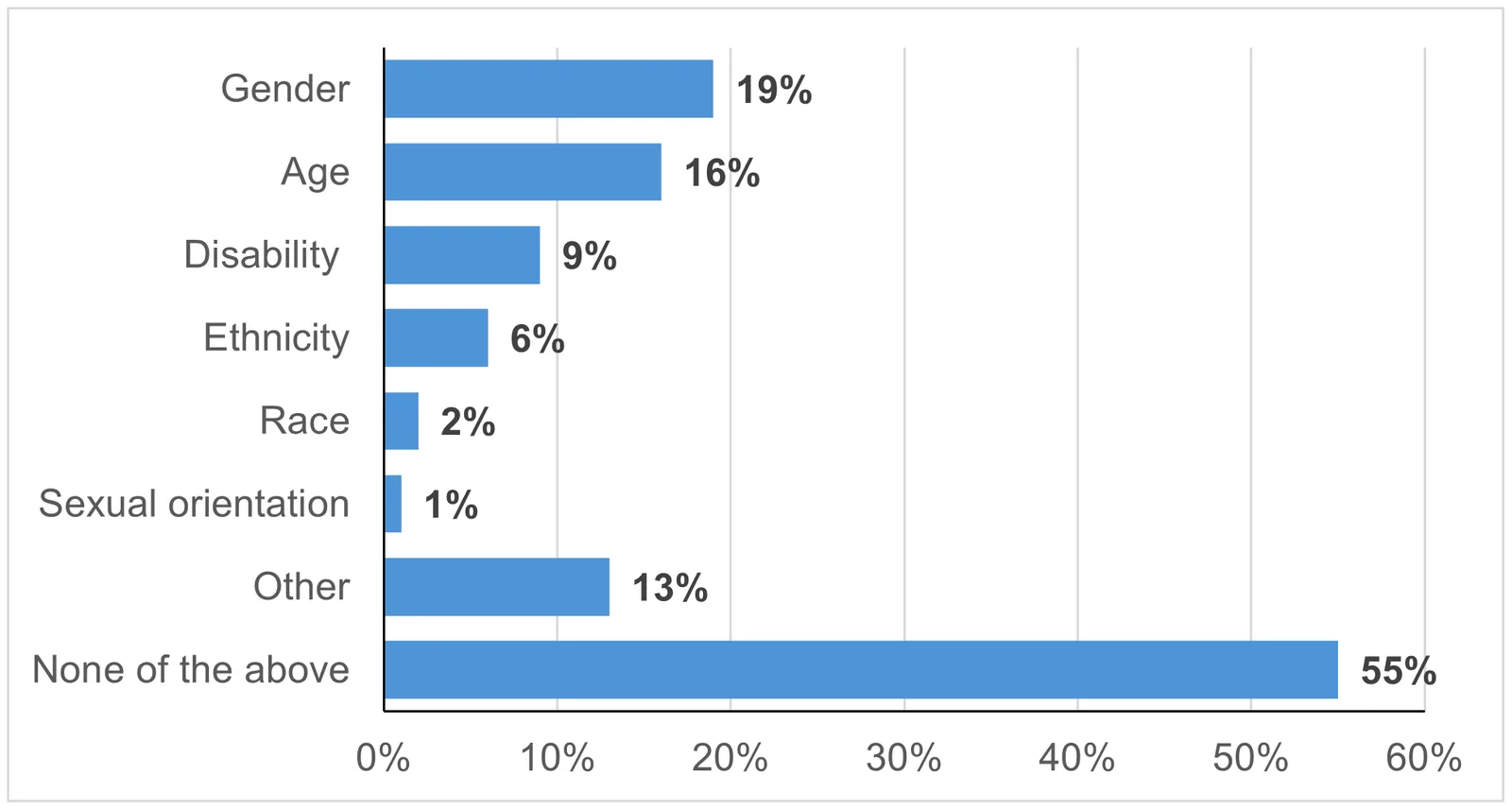
Proportion of participants reporting that aspects of their identity have negatively impacted their research career or experience within the research community *Participants were asked to select all that apply.

Overall, 45% of participants reported that aspects of their identity negatively impacted their research career, in particular, gender (19%), age (16%), and disability (9%) (Fig 8).

Of those who viewed gender as a barrier to career progression, nearly all were women (n = 47 of 49), highlighting a significant gender disparity. Family and caring responsibilities further increased these challenges.

*“I've recently returned from maternity leave and my leave overlapped with a key funding call in my research area that I could not apply to unfortunately. I have also missed opportunities for promotion and now my university has paused all promotions probably for two years. I'm now working part time which I'm happy with but is inevitably detrimental to my career in the short to medium term. (ID125, Assistant Professor/Lecturer, Part-time, Permanent, Social Science)*

Age related discrimination was reported by both older and younger researchers, most commonly among those aged 55–64 and 18–34. In particular, participants who entered academia later on in their career, which was often the case for participants in our sample, described difficulty in securing early-career funding.

*"Moving into academia later (from a related but different field) made it hard to gain PhD funding/initial grants..." (ID101, Assistant Professor/Lecturer, Full-time, Fixed-term, Other discipline)*

Among 37 participants who reported that they had a disability**,** more than half (n = 23) reported that this had negatively impacted their research career progression. For some this was due to difficulties conducting certain research activities.

*“I’m neurodiverse and I struggle hugely with the social aspects of research (conferences, research groups) as well as remaining focused on research.” (ID16, Research Assistant or equivalent, Other contract type, Education).*

Stronger institutional support, such as improved visibility and inclusion of diverse populations within universities and national research organisations, was seen as essential for more inclusive and equitable research workforce. In addition, broadening the scope of research topics to include diverse populations was seen as a way to attract and support a more ethnically diverse research workforce.

*“Diversity of topics explored within [PEOLC] research encompassing diverse populations to encourage a diversity of researchers.” (ID206, Research Assistant, Full-time, Fixed-term, Nursing)*

## Discussion

Our findings reveal that palliative care researchers are positive about many aspects of their working environment; and report a collaborative working culture, which values creativity and rigour. Indeed, when compared to the perspectives of researchers across a range of fields [19,20], those involved in palliative care research are generally more positive about nearly all aspects of their working environment. However, while most indicate that they wish to remain active in PEOLC research, only two-thirds believe they are likely to continue working in this area in the next five years. Lack of funding was most frequently identified as a barrier to a successful palliative care research career. For those on fixed term contracts, job insecurity was described as a significant barrier to career progression and remaining in the field. This coexistence of positive and collaborative working environments with high levels of insecurity is not necessarily contradictory. Nor is the gap between researchers’ strong desire to remain in PEOLC research and their lower expectation of being able to do so. Supportive colleagues and a positive, collaborative team culture may improve researchers’ individual experiences. However, structural problems such as lack of funding, job insecurity, and unclear career pathways persist beyond the control of individual researchers and research teams. To sustain and grow palliative care research, there is a need for action at organisational, community, policy and societal levels. At a broad level this includes greater access to funding for researchers at all career stages; increased visibility of palliative care as a vital research area; greater collaboration and networking opportunities, protected research time and further efforts to enhance research workforce diversity.

Action is needed to increase visibility of PEOLC research. Palliative care researchers need to engage more with policy-makers, service commissioners, health and social care managers, educators and funders; and demonstrate how their findings can inform decision-making related to health service delivery. In identifying key partners, researchers might consider: ‘*who can act on the basis of our research knowledge*’ and ‘*who can influence those who can act’* [21]. Engaging with key partners early on in the research process, and involving them in research facilitates knowledge exchange and impact [22–24]. Introducing palliative care to students (medicine, nursing, allied health professionals, psychologists) early on in their training also raises visibility and understanding of the field. In the UK, palliative care has increasingly been introduced into the medical school curriculum [25,26], but undergraduate training in palliative care in many relevant fields such as nursing and psychology remains underdeveloped [27–29]. Education can also play a role to reduce societal misperceptions about palliative care and discomfort around death and dying [30–32]. A public health approach is recommended to increase visibility of palliative care, and encourage conversations around death and dying [33]. The PEOLC research community is well placed to advocate for greater openness around death and dying and bereavement across the UK.

Funding constraints are a recognised challenge to research growth and career progression in other fields [20]. However, our findings reflect those of other analyses pointing to significant underinvestment in palliative care research compared to other areas [4,5]. Limited government funding for the palliative care sector, and a reliance on charities to provide essential services [34] creates financial instability. Consequently, organisations with a remit to provide palliative care prioritise core clinical service delivery, which constrains investment in research infrastructure and protected staff time, thereby limiting overall research capacity. Government research funders typically prioritise research which prevents ill health and helps people to remain healthy and fit for work which makes it harder for palliative care researchers to compete for funding [35]. The multidisciplinary and holistic nature of palliative care can make intervention research more complex, which may affect its attractiveness to funders [17]. ‘Highlight notices’ or ring-fenced funding for palliative care are essential to ensure those conducting palliative care research can compete for funding and progress the field. The National Institute for Health and Care Research in the UK has highlighted palliative care as a priority area for many of its research programmes [36], which should help increase research activity and build capacity in the coming years.

Fellowships for individual researchers were identified as the most needed type of funding. Fellowships are associated with increased academic output, skills development, career progression, and are an increasingly important part of the funding landscape internationally [37–41]. In the UK, Marie Curie, a third-sector organisation providing specialist palliative care, has funded several research fellowships, including that of the corresponding author (AF) [42]. However, increased fellowship opportunities for early- and mid-career researchers are needed, alongside training in fellowship-writing skills to improve the competitiveness of palliative care applications.

After fellowships, participants most frequently selected ‘large research centre grants’ as essential to grow PEOLC research capacity. Research centres of excellence can act as hubs that increase regional research capacity, support researcher skills development, and enable mentoring, collaboration and knowledge exchange [43–46]. Large centres with core funding rely less on short-term grants; therefore, have better continuity and greater capacity to apply for funding for ambitious high-quality research programmes. Early-career researchers have more opportunities to thrive in research groups with sufficient critical mass, where they can benefit from mentorship, peer-support and collaboration opportunities for future work. Given the multidisciplinary nature of palliative care, centres that bring together researchers and stakeholders from a variety of disciplines and backgrounds are particularly well-suited to address contemporary challenges associated with meeting the palliative care needs of an ageing population [47]. Research centres that integrate palliative care with adjacent fields such as geriatrics, chronic illness, and social care can generate more holistic and coordinated approaches to meet the needs of ageing societies.

Investment in palliative care research and knowledge exchange networks is essential to grow the field and has been recommended in the past [7,9,48]. Networks provide platforms where researchers, healthcare professionals and a diverse range of stakeholders come together to share expertise, knowledge and opportunities [46]. Such networks are important for developing ideas, and creating conditions for research creativity and innovation [49]. The more highly connected the network is, the more power it has to facilitate knowledge flow and cross-fertilisation of ideas. In the UK, recently established networks such as the Palliative Care Research Incubator, and the Mental Health and Wellbeing in Advanced Illness Network have been established to help build capacity and increase visibility of the palliative care research [50,51]. Continued investment in such initiatives alongside funding to develop local and/or regional networks to support research collaboration in specified geographic areas, could play a vital role in progressing palliative care research that informs service development and addresses the priorities of local populations.

Lack of time and unmanageable workloads were frequently identified as a barrier to research activity. Time constraints are a challenge for those wishing to become involved in research in many areas [52–56]; however, this may be heightened in palliative care. For example, a 2021 UK survey found that only 35% of palliative medicine specialist trainees felt adequately supported to conduct research, and many conducted research in their own time [11]. A subsequent national online survey of palliative medicine consultants found that while over three-quarters were interested in conducting research, 83% had no allocated time in their job plan for this [57]. There are few academic posts and structured academic career pathways within the palliative care specialty, in contrast to fields such as oncology or cardiology where research infrastructure is well established [12,58]. This may be because palliative care is less well-established than other fields, and palliative care teams may be small and fragmented, working across several clinical areas. For this reason, it has been argued that palliative medicine may need to be considered a special case, in need of additional support to build research capacity and infrastructure [57]. Nurses and allied health professionals also find it hard to find the time to get involved in palliative care research, and were most likely to report ‘lack of opportunities’ as a barrier to research in our study, despite the expertise they bring [59]. Research governance and recruitment processes are particularly time consuming due to factors such as participant frailty and distress – less experienced researchers needs sufficient time and support to navigate these processes. Engaging clinicians in research is associated with improved healthcare outcomes [60] and protecting time for research can benefit patients and families in the longer-term. Greater opportunities for clinicians to link with palliative care research centres and networks are needed, as it is impossible to progress most research projects working in isolation.

Nearly half of participants reported experiencing discrimination related to their identity, such as gender and age. However, levels were much lower compared to those reported in the Wellcome Trust research cultures survey [6]. Internationally, women are underrepresented in research compared to men, however, in recent years there have been increases in representation of female researchers in nursing, medicine and psychology – disciplines closely linked with palliative care [61], which may partly account for the lower levels of gender related barriers to progression we found. Despite this, gender was still a barrier for nearly one-in-five who responded to our survey. The qualitative data highlighted structural challenges, such as career breaks due to maternity leave, as having a negative impact on progression. Precarious employment may disproportionally affect researchers with caring responsibility by disrupting employment continuity and making it more difficult to remain in research following a period of leave [62]. Age was reported as a source of discrimination, though the proportion of those reporting discrimination due to age was lower than what has been reported elsewhere [6], with both older and younger workers reporting age-related barriers to career progression. Age-related inequalities in access to funding were also identified in the qualitative findings. Researchers entering academia later in their careers may face challenges accessing funding opportunities which are designed around conventional trajectories. Although such researchers may be at an early stage of their academic careers, they may not fit easily within eligibility criteria or expectations that assume linear progression through academic training. Researchers with disabilities may also be disadvantaged by prevailing expectations of continuous output, success in securing funding, and linear career progression. These findings suggest that current research structures may unintentionally disadvantage those whose career pathways or personal circumstances do not align with conventional academic progression. This may reduce workforce diversity and narrow the scope of research topics required to ensure PEOLC research reflects the needs of diverse populations.

The proportion of participants reporting discrimination due to race or ethnicity was lower compared with the Wellcome Trust survey, however 85% of respondents described themselves as ‘White’, slightly higher than the national average for England and Wales (82%), suggesting slightly less diversity in palliative care research compared to the country as a whole. In the UK, the National Institute for Health and Care Research’s inclusion strategy 2022–2027 [63] commits to quality, diversity and inclusion, emphasizing the importance of developing researchers from multiple disciplines, geographies and backgrounds, and addressing barriers to career progression arising from personal characteristics. Diverse research teams that partner with the communities they serve are needed to prioritise research that enhances equity and diversity in palliative care practice [64].

### Strengths and limitations

A strength of our study was that we were able to compare our findings with a previously conducted survey of researchers in the UK [6], which allowed us to see how perceptions of the palliative care research context are similar to, and differ from, perceptions of the wider research context. Another strength was that we employed a mixed-methods design, with qualitative findings enabling contextualisation and expansion of quantitative findings. This integration provided a more detailed understanding of the factors shaping the PEOLC research environment and workforce. However, the qualitative depth may have been constrained by the data collection method with a reliance on open-ended survey responses rather than in-depth interviews or focus groups. This limitation is typical of the ‘questionnaire variant’ of convergent mixed method designs, where the qualitative component is often an add-on to the quantitative elements [14].

We were unable to calculate a response rate, as our survey was disseminated on social media and via a range of networks, making it impossible to determine how many individuals saw the invitation. There may be a degree of self-selection bias, where only those having spare time to respond to the survey, or those already linked in with research networks were more likely to participate, with isolated researchers and clinical academics being underrepresented. We had broad inclusion criteria, as we wanted to include all those involved in conducting palliative care research, irrespective of their role or location. While this was a strength, it was also a possible weakness in that we also included those for whom conducting palliative care research is only a small part of their role.

### Implications

Our findings highlight the challenges to sustaining and growing PEOLC research activity in the UK. We have identified a need for (i) greater visibility of PEOLC as a vital research field; (ii) increased funding to support those undertaking PEOLC research, across career stages and disciplines; (iii) increased opportunities for collaboration and networking; (iv) protected research time, and (v) support to build a more diverse research community. Drawing on our findings, we summarise some the actions that could be taken by research funders, research leaders and anyone involved in palliative care research, to sustain and grow the field, ensuring that future policy, service development and clinical practice continues to be informed by high-quality evidence. (Table 2)

**Table 2:** Strategies to sustain and grow PEOLC research activity.

Audience	Proposed strategies
**Research funders**	1. Introduce ‘highlight notices’ or targeted calls for PEOLC research to sustain and grow activity. 2. Increase funding for PEOLC research fellowships linked to tenure-track positions. 3. Fund multidisciplinary PEOLC research centres with longer-term core funding. 4. Increase funding for research networks to enhance opportunities for collaboration. 5. Increase small grant and seed funding opportunities. 6. Create more funding opportunities to enable protected research time for clinically based researchers. 7. Ensure funding welcomes researchers on non-linear career paths and those working in a part-time capacity. 8. Create bridging funding to support researchers between grants or positions. 9. Increase funding opportunities targeting nurses to support research involvement and leadership. 10. Create funding opportunities for PEOLC research to inform healthcare policy and strategy development.
**Research leaders**	1. Seek opportunities for engagement and impact activities to demonstrate the impact of PEOLC research. 2. Support more structured and flexible career pathways, especially for clinical academics. 3. Seek funding for administrative support associated with research grant applications. 4. Ensure training opportunities and support for early-career researchers in research governance. 5. Help shift institutional attitudes to appreciate the contributions of contract researchers. 6. Build a diverse team of researchers with varied lived experiences. 7. Proactively create opportunities for networking and collaboration to support early-career researchers. 8. Create opportunities for mentorship both within and outside of host institutions. 9. Introduce palliative care to students in relevant disciplines at an early stage to raise awareness of the field. 10. Support multidisciplinary research groups with sufficient critical mass to support local research collaboration and knowledge exchange.
**All researchers**	1. Harness opportunities to advocate for the field and raise awareness of its importance. 2. Seek opportunities to join a palliative care research network or group.

## Conclusion

Despite a commitment to PEOLC research, and a positive view on many aspects of their work environment, many palliative care researchers believe they are unlikely to remain in the field in the next five years. To sustain current levels of activity, and build future capacity there is a need for increased research project funding, greater access to fellowships, alongside investment in large research centres or networks. Given the multidisciplinary nature of palliative care, greater opportunities for collaboration and networking are needed to allow researchers to connect, share knowledge and skills, and develop competitive research funding applications. Protected research time is essential for clinicians undertaking research; alongside access to academic teams. A focus on building a diverse research community that understands the population it serves will ensure that issues around equity and access for all are prioritised. Finally, by increasing engagement and research impact activities, and advocating for palliative care, the research community can help increase visibility of palliative care as a vital area of research, essential for meeting the health and social care needs of an ageing population.

## Supporting information

S1 File Survey. Welcome to our survey.
(PDF)

S2 File Search. Search for palliative care researchers.
(DOCX)

S1 Table Participant Characteristics. (DOCX)

S1 Fig Lack of funding as a barrier by role. (DOCX)

S2 Fig Lack of funding as a barrier by research discipline. (DOCX)

S1 Data Anonymised Quantitative Data. (XLSX)

S2 Data Anonymised Qualitative Data. (XLSX)
