# Supplementary material for: Growing palliative and end-of-life care research in the United Kingdom: Findings from a mixed methods survey
Source: PLoS One. 2026 Sep 15;21(9):e0357024. doi: 10.1371/journal.pone.0357024 (PMC13577371; doi:10.1371/journal.pone.0357024)
Supplement: Supplementary file 1 [file pone.0357024.s001.pdf]

S1 Appendix. Online questionnaire

Welcome to our survey!

This survey includes both multiple-choice and open-ended questions. To maintain the anonymity of your responses, please avoid entering any personal or identifiable information, such as names or locations. All responses will be reviewed, and if any identifiable information is detected, it will be anonymised prior to data analysis.

If you have any questions, contact Ikumi at iokamoto@ed.ac.uk.

Closing date: Sunday 12th January 2025

Thanks again for your help!

10. **Which of the following best describes your current role?** *

- ◯ Clinical Researcher/Clinical Academic/Clinical Research Training Fellow or equivalent
- ◯ Professor/Group Leader/Director/Head of Department or equivalent
- ◯ Associate Professor/Senior Lecturer/Reader or equivalent
- ◯ Assistant Professor/Lecturer or equivalent
- ◯ Senior Research Fellow/Research Fellow/Senior Research Associate/Research Associate or equivalent
- ◯ Research Assistant or equivalent
- ◯ Research Manager/Research Lead/Research Facilitator or equivalent
- ◯ Research Nurse or equivalent
- ◯ PhD Student
- ◯ Prefer not to say
- ◯ Other (e.g., Retired; Honorary; Research Technician; or have multiple roles)

11. **If you selected 'other', please elaborate here.**

12. **If you are employed, which of the following best describes your overall current employment status?** *

- ◯ Full-time
- ◯ Part-time
- ◯ Not applicable

◯ Other

13. **If you selected 'other', please specify.**

14. **What type of contract do you have <u>in relation to research work in general</u>?** *

◯ Permanent contract
◯ Fixed-term contract
◯ Don't know
◯ Other

15. **If you selected 'other', please specify.**

16. **If you have a fixed-term research contract, what is the total length of <u>your current research contract, overall</u>?** *

◯ Up to 1 year
◯ More than 1 year to 2 years
◯ More than 2 year to 3 years
◯ More than 3 year to 5 years
◯ More than 5 years
◯ Unsure
◯ Other

17. **If you selected 'other', please specify.**

18. **How many hours per week are you paid to spend on research activities overall?** *

- None
- 1
- 2
- 3
- 4
- 5
- 6
- 7
- 8
- 9
- 10
- 11
- 12
- 13
- 14
- 15
- 16
- 17
- 18
- 19
- 20
- 21
- 22
- 23
- 24
- 25
- 26
- 27
- 28

- ◯ 29
- ◯ 30
- ◯ 31
- ◯ 32
- ◯ 33
- ◯ 34
- ◯ 35
- ◯ 36
- ◯ 37
- ◯ 38
- ◯ 39
- ◯ 40
- ◯ 41
- ◯ 42
- ◯ 43
- ◯ 44
- ◯ 45
- ◯ 46
- ◯ 47
- ◯ 48
- ◯ 49
- ◯ 50

19. **On average, roughly how much of your work time is devoted to <u>research on palliative and end-of-life care</u>?** *

- ◯ All my work time (100%)
- ◯ About half of my work time or more (50% or more than 50%)
- ◯ Less than half of my work time (less than 50%)
- ◯ None of my working week (free time only)
- ◯ Not applicable
- ◯ Other

20. **If you selected 'other', please specify.**

21. **Roughly, how many years have you been involved in palliative and end-of-life care research in total (including PhD time)?** *

- ◯ Less than 1 year
- ◯ 1 year to less than 5 years
- ◯ 5 years to less than 10 years
- ◯ 10 years to less than 20 years
- ◯ 20 years or more
- ◯ Prefer not to say
- ◯ Other

22. **If you selected 'other', please specify.**

23. **Which of the following describes your research discipline or sector?**
If you have more than one research discipline, please indicate your primary discipline here, and your secondary discipline in Question 25. *

- ◯ Medicine
- ◯ Education
- ◯ Nursing
- ◯ Health Economics
- ◯ Statistics
- ◯ Allied Health Profession
- ◯ Psychology
- ◯ Social Science (excluding psychology)
- ◯ Social Work
- ◯ Health Sciences
- ◯ Humanities

◯ Pharmacy

◯ Public Health

◯ Other

24. **If you selected 'other', please specify.**

25. **If you have more than one research discipline, please indicate your <u>secondary</u> discipline here (or choose 'no secondary discipline').** *

◯ No secondary discipline

◯ Medicine

◯ Education

◯ Nursing

◯ Health economics

◯ Statistics

◯ Allied health profession

◯ Psychology

◯ Social Sciences (excluding psychology)

◯ Social Work

◯ Health Sciences

◯ Humanities

◯ Pharmacy

◯ Public Health

◯ Other

26. **If you selected 'other', please specify.**

27. **Which <u>best</u> describes your current workplace?**
If on leave, please indicate the workplace you intend to return to if applicable.
If you have more than one workplace, please indicate your <u>primary</u> workplace here and your secondary workplace in Question 29. *

- ◯ University
- ◯ Not-for-profit research Insititute (e.g., charity-funded)
- ◯ Research council
- ◯ Hospital
- ◯ Hospice
- ◯ General practice
- ◯ Community
- ◯ Care home
- ◯ Pharmacy
- ◯ Industry (e.g., pharmaceuticals, medical technology)
- ◯ Other

28. **If you selected 'other', please specify.**

29. **If you have more than one workplace, please indicate your <u>secondary</u> workplace here (or choose 'no secondary workplace'). If on leave, please indicate the workplace you intend to return to if applicable.** *

- ◯ No secondary workplace
- ◯ University
- ◯ Not-for-profit research Insititute (e.g., charity-funded)
- ◯ Research council
- ◯ Hospital

- ◯ Hospice
- ◯ General practice
- ◯ Community
- ◯ Care home
- ◯ Pharmacy
- ◯ Industry (e.g., pharmaceuticals, medical technology)
- ◯ Other

30. **If you selected 'other', please specify.**

31. **Please indicate which of the following palliative and end-of-life care domains is your research primarily focused on?** *

- ◯ Physical (e.g., pain/symptom management)
- ◯ Psychological (e.g., mental wellbeing, emotional support, anxiety and depression)
- ◯ Social (e.g., palliative/end-of-life care of underrepresented groups, financial/legal issues, access to care)
- ◯ Spiritual (e.g., meaning in their experiences, religious practices, spiritual counselling)
- ◯ Other

32. **If you selected 'other', please specify.**

33. **If your palliative and end-of-life care research is focused on people with specific conditions, please indicate the relevant area.** *

- ◯ Cancer
- ◯ Organ failure (e.g., heart failure, kidney/liver failure, COPD)
- ◯ Neurological disorders
- ◯ Multimorbidity

- ◯ Frailty and/or dementia
- ◯ Not applicable
- ◯ Other

34. **If you selected 'other', please specify.**

35. **Please indicate up to five research areas in which you are currently most active.** *

- ☐ Assisted dying/Euthanasia
- ☐ Bereavement/grief
- ☐ Complementary therapies
- ☐ Education
- ☐ Epidemiology
- ☐ Ethics
- ☐ Family & carer support
- ☐ Health economics
- ☐ Identification for a palliative approach
- ☐ Information / Communication
- ☐ Methodology
- ☐ Outcome measures/ tools
- ☐ Out-of-hours
- ☐ Paediatric palliative care
- ☐ Palliative care for non-malignant conditions
- ☐ Palliative care service delivery & organisation
- ☐ Palliative care in non-UK societies/Global palliative care
- ☐ Paramedics
- ☐ Physical symptoms
- ☐ Policy
- ☐ Primary care
- ☐ Psychological support and interventions
- ☐ Public health
- ☐ Rehabilitation

- [ ] Social care
- [ ] Spiritual care
- [ ] Supportive care
- [ ] Other

36. **If you selected 'other', please specify.**

37. **Within the past five years, have you been awarded funding as a Chief or Principal Investigator leading a palliative and end-of-life research project?** *

◯ Yes
◯ No

38. **If yes, approximately what was the total funding amount for all grants related to palliative and end-of-life care for which you were the Chief or Principal Investigator in the last five years?** *

◯ Over £1 million
◯ £750k - £1 millions
◯ £500,000 - £749,999
◯ £250,000- £499,999
◯ £100,000 - £249,999
◯ £50,000 to under £100,000
◯ £10,000 to just under £50,000
◯ Under £10,000

39. **Approximately what proportion of your total Chief Investigator or Principal Investigator grant income does palliative and end-of-life care research represent?** *

◯ Less than 25%
◯ 26% to 50%
◯ 51% to 75%
◯ 76% to 100%

40. **How are your current palliative and end-of-life care research projects funded? (Tick all that apply)** *

☐ University
☐ Charity
☐ Research Council or Government (e.g., ESRC, NIHR)
☐ Hospital
☐ Pharmaceutical companies
☐ Prefer not to say
☐ Not applicable

- [ ] Other

41. **If you selected 'other', please specify.**

42. **What type of funding is most needed to ensure the growth of palliative and end-of-life care research activity across the UK? Please select up to three.** *

- [ ] Large research centre grants
- [ ] Research Fellowships for independent researchers
- [ ] Grants to support clinicians involved in research
- [ ] Seed funding for new research /developmental studies
- [ ] Funding of academic positions – Lecturer/Senior lecturer
- [ ] Funding of senior academic positions – Reader/ Professor
- [ ] Small grants for independent researchers
- [ ] Conference travel grants
- [ ] Research Nurse funding
- [ ] Other

43. **If you selected 'other', please specify.**

44. **To what extent do you agree or disagree with the following statements relating to your current working environment?** *

My working environment promotes a good work-life balance

- Strongly disagree
- Disagree
- Neither disagree nor agree
- Agree
- Strongly agree
- Not Applicable

My working environment promotes a collaborative culture

- Strongly disagree
- Disagree
- Neither disagree nor agree
- Agree
- Strongly agree
- Not Applicable

Creativity is welcomed within my working environment in all its forms

- Strongly disagree
- Disagree
- Neither disagree nor agree
- Agree
- Strongly agree
- Not Applicable

Healthy competition is encouraged within my working environment

- Strongly disagree
- Disagree
- Neither disagree nor agree
- Agree
- Strongly agree
- Not Applicable

Unhealthy competition is present within my working environment

- Strongly disagree
- Disagree
- Neither disagree nor agree
- Agree

- Strongly agree
- Not Applicable

My institution/workplace values speed of results over quality

- Strongly disagree
- Disagree
- Neither disagree nor agree
- Agree
- Strongly agree
- Not Applicable

My institution/workplace could do more to ensure research practices do not cut corners

- Strongly disagree
- Disagree
- Neither disagree nor agree
- Agree
- Strongly agree
- Not Applicable

Rigour of results is considered an important research outcome by my institution/workplace

- Strongly disagree
- Disagree
- Neither disagree nor agree
- Agree
- Strongly agree
- Not Applicable

My institution/workplace places more value on meeting metrics, than it does on research quality

- Strongly disagree
- Disagree
- Neither disagree nor agree
- Agree
- Strongly agree
- Not Applicable

I am confident that my institution/workplace would listen and take action if I raised a concern

- Strongly disagree
- Disagree
- Neither disagree nor agree
- Agree

- ◯ Strongly agree
- ◯ Not Applicable

The culture around research in my working environment supports my ability to do good quality research

- ◯ Strongly disagree
- ◯ Disagree
- ◯ Neither disagree nor agree
- ◯ Agree
- ◯ Strongly agree
- ◯ Not Applicable

My institution/workplace's expectations of me to undertake a number of roles leaves me little time for research

- ◯ Strongly disagree
- ◯ Disagree
- ◯ Neither disagree nor agree
- ◯ Agree
- ◯ Strongly agree
- ◯ Not Applicable

My working environment hinders researchers getting on with their research

- ◯ Strongly disagree
- ◯ Disagree
- ◯ Neither disagree nor agree
- ◯ Agree
- ◯ Strongly agree
- ◯ Not Applicable

My institution/workplace provides me with support to navigate the grant application process

- ◯ Strongly disagree
- ◯ Disagree
- ◯ Neither disagree nor agree
- ◯ Agree
- ◯ Strongly agree
- ◯ Not Applicable

45. **Do you face or have you faced any barriers to achieving a successful career in palliative and end-of-life care research? (Tick all that apply)** *

- ☐ Lack of opportunities
- ☐ Lack of support from institution/workplace

- [ ] Unmanageable workload
- [ ] Job insecurity
- [ ] Lack of funding
- [ ] Bullying and harassment
- [ ] Lack of training in relevant field
- [ ] Lack of training in relevant skills
- [ ] Caring responsibilities
- [ ] Inability to relocate
- [ ] Prejudice and discrimination
- [ ] Lack of advice and guidance
- [ ] Lack of flexibility (remote work/working hours)
- [ ] None of the above
- [ ] Other

46. **If you selected 'other' please specify.**

47. **To what extent do you agree or disagree with the following statements?** *

I wish to remain actively involved in conducting palliative and end-of-life care research in the next 5 years

- ○ Strongly disagree
- ○ Disagree
- ○ Neither disagree nor agree
- ○ Agree
- ○ Strongly agree
- ○ Don't know

I am likely to remain active in palliative and end-of-life care research in the next 5 years.

- ○ Strongly disagree
- ○ Disagree
- ○ Neither disagree nor agree
- ○ Agree
- ○ Strongly agree
- ○ Don't know

48. **What do you perceive to be the main barriers to you to remaining active in palliative and end-of-life care research?**

49. **What would most help you to remain active in palliative and end-of-life care research in the future? Please be as specific as possible.**

50. **What would most help and encourage you or new researchers to join the field of palliative and end-of-life care research?**

51. **Please suggest any measures that could improve career progression and opportunities for all within palliative and end-of-life care research, focusing on inclusion, equity, and equal access.**

52. **Are there any more comments you'd like to share with us?**

About you

We have a few additional questions to help us better understand the palliative and end-of-life care research workforce and to identify potential gaps or underrepresented groups in the field.

As part of this, we are also asking questions related to your identity, such as ethnicity and sexual orientation. To ensure your privacy, we will not share information like roles and regions alongside protected characteristics outside the research team, preventing the identification of individuals from minority groups.

If you do not feel comfortable providing this information, please select "Prefer not to say". Please be assured that your answers are strictly confidential, and this survey is not linked to any identifiable data, such as your name or email address.

For further information about data privacy for research participants please refer to:
https://data-protection.ed.ac.uk/privacy-notice-research

53. **Which of the following best describes your gender?** *

◯ Female
◯ Male
◯ Non-binary
◯ Prefer to self-describe
◯ Prefer not to say

54. **If you selected 'Prefer to self-describe', please specify if possible.**

55. **Is your gender the same as the sex you were assigned at birth?** *

◯ Yes
◯ No
◯ Prefer not to say

56. **Which of the following best describes your sexual orientation?** *

◯ Asexual
◯ Bi/bisexual

- ◯ Gay man
- ◯ Gay woman/lesbian
- ◯ Heterosexual/straight
- ◯ Queer
- ◯ Prefer not to say
- ◯ Prefer to self-describe

57. **If you selected 'Prefer to self-describe', please specify if possible.**

58. **How old are you?** *

- ◯ 18-24
- ◯ 25-34
- ◯ 35-44
- ◯ 45-54
- ◯ 55-64
- ◯ 65-74
- ◯ 75+
- ◯ Prefer not to say

59. **Which of the following best describes you?** *

- ◯ Asian / Asian British: Bangladeshi
- ◯ Asian / Asian British: Indian
- ◯ Asian / Asian British: Pakistani
- ◯ Asian / Asian British: Chinese
- ◯ Any other Asian background
- ◯ Black / Black British: African
- ◯ Black / Black British: Caribbean
- ◯ Any other Black background
- ◯ Mixed / multiple ethnic groups: Asian and White
- ◯ Mixed / multiple ethnic groups: Black Caribbean and White
- ◯ Mixed / multiple ethnic groups: Black African and White

- ◯ Any other mixed / multiple ethnicity background
- ◯ White: English / Welsh / Scottish / Northern Irish / British
- ◯ White: Irish
- ◯ White: Gypsy or Irish Traveller
- ◯ Any other White background
- ◯ Arab
- ◯ Any other ethnic background
- ◯ Prefer not to say
- ◯ Unknown

60. **Do you feel that any of the following aspects of your identity have negatively impacted your research career or your experience within the research community? (Tick all that apply)** *

- ☐ Age
- ☐ Gender
- ☐ Sexual orientation
- ☐ Disability
- ☐ Race
- ☐ Ethnicity
- ☐ None of the above
- ☐ Other

61. **If you feel able to, please share details below. (Please avoid including any personally identifiable information)**

62. **Where is your main workplace based?** *

- ◯ Wales
- ◯ Scotland
- ◯ Northern Ireland
- ◯ England

◯ Prefer not to say

63. **Do you have any caring responsibilities on a daily/weekly basis? (Tick all that apply)** *

☐ None
☐ Primary carer of a child/children (under 18)
☐ Primary carer of disabled child/children
☐ Primary carer or assistant for a disabled adult (18 years or over)
☐ Primary carer or assistant for an older person/people (65 and over)
☐ Secondary carer (another person carries out the main caring role)
☐ Prefer not to say
☐ Other

64. **If you selected 'other', please specify if possible.**

65. **Were you first generation or first in family to attend university?** *

◯ Yes
◯ No
◯ Unsure/Not applicable
◯ Prefer not to say

66. **Do you consider yourself to be a disabled person?** *

◯ Yes
◯ No
◯ Prefer not to say

67. **If possible, please outline brief details of your disability.**

Thank you very much for taking the time to complete this survey.

The study's findings will be widely disseminated through various channels, including a project report to Marie Curie and peer-reviewed academic journal articles, to raise awareness of the need for increased PEOLC research investment, highlighting funding gaps, challenges and opportunities to enhance future research capacity in the UK.

After you submit the survey, you will see a new page with a link to click if you would like to stay in touch with us, receive funding information from Marie Curie, and/or enter for a chance to win an Amazon voucher.
