# Supplementary material for: Growing palliative and end-of-life care research in the United Kingdom: Findings from a mixed methods survey
Source: PLoS One. 2026 Sep 15;21(9):e0357024. doi: 10.1371/journal.pone.0357024 (PMC13577371; doi:10.1371/journal.pone.0357024)
Supplement: Supplementary file 2 [file pone.0357024.s008.pdf]

The manuscript addresses a relevant and underexplored topic concerning the sustainability and development of the palliative and end-of-life care (PEOLC) research workforce in the United Kingdom. The study presents potentially important findings regarding funding, researcher retention, and research capacity building in the field. The manuscript is generally well structured and has scientific merit. However, some methodological and analytical aspects require further clarification and development prior to publication.

MAJOR COMMENTS

1. The manuscript states that it employed a convergent mixed methods design ("questionnaire variant of the convergent mixed methods research design"). However, the integration between the quantitative and qualitative components is insufficiently articulated throughout the manuscript. Currently, the qualitative findings mainly serve as illustrations of the quantitative results. The authors should clarify: (i) at which stages integration occurred; (ii) how qualitative findings modified, deepened, or explained the quantitative findings; (iii) which conclusions were only possible due to the combination of methods. The statement: "Quantitative and qualitative data were then synthesized and consolidated through iterative discussions within the research team" is overly broad for a mixed methods study. Greater methodological detail regarding the integration process is recommended.

2. The authors state that the design allowed for "enriching the overall findings," but this is insufficient as a methodological rationale. The manuscript would benefit from explicitly clarifying: (1) why quantitative data alone would have been insufficient; (2) why qualitative data were necessary; (3) what the intended purpose of integration was (e.g., complementarity, expansion, triangulation, development). Without this clarification, the study reads more as a survey containing open-ended questions than as a fully integrated mixed methods study.

3. The "Strengths and limitations" section partially overstates the depth of methodological integration. The authors state: "the integration of quantitative and qualitative data allowed for a rich understanding" However, given that the qualitative data derive from open-ended online survey responses, the interpretive depth is inherently more limited than would be expected from interviews or focus groups. The authors are encouraged to moderate this statement and explicitly acknowledge that: (a) the design enabled contextualization of the quantitative findings; (b) however, the qualitative depth was constrained by the nature of the data collection method.

4. The manuscript could better explore divergent findings or tensions across datasets. For example, participants reported collaborative and positive working environments, while simultaneously reporting high levels of job insecurity, workload burden, and likelihood of leaving the field. This contradiction is analytically interesting and deserves deeper exploration in the discussion section: How can positive work culture coexist with substantial precarity? Is there a strong professional or moral commitment sustaining engagement despite instability? Is academic precarity becoming normalized within the field? At present, these tensions are described but not sufficiently interpreted.

5. The manuscript describes inequalities related to gender, age, and disability, but the discussion remains largely descriptive. The authors are encouraged to further explore: (a)

how precarious employment may disproportionately affect women and researchers with caring responsibilities; (b) how late academic entry pathways affect access to funding; (c) how prevailing productivity models in academia may reproduce structural inequities. This would strengthen the manuscript’s critical contribution.

MINOR COMMENTS

6. Figure 4 provides a useful visual summary of factors influencing workforce sustainability. However, the manuscript would benefit from a clearer explanation in the Methods section regarding how the five themes were operationally derived through integration of quantitative and qualitative findings.

7. It would be useful to report more clearly:
- how many participants responded to the open-ended questions;
- the average length of qualitative responses;
- the overall volume of qualitative material analyzed.

These details would help readers assess the robustness of the qualitative component.

Overall recommendation

The study addresses an important issue and offers a potentially valuable contribution to strengthening palliative care research capacity. However, the manuscript requires greater methodological clarity regarding the mixed methods design, particularly in relation to data integration and the inferential contribution generated by combining methods.
