# Supplementary material for: Growing palliative and end-of-life care research in the United Kingdom: Findings from a mixed methods survey
Source: PLoS One. 2026 Sep 15;21(9):e0357024. doi: 10.1371/journal.pone.0357024 (PMC13577371; doi:10.1371/journal.pone.0357024)
Supplement: Supplementary file 3 [file pone.0357024.s002.docx]

**S2 Appendix. Website search approach**

A website trawl of all UK universities was conducted from May to November 2024 to identify researchers involved in palliative and end-of-life care research.

Search terms used for the website trawl:

- Palliative
- End of life
- Hospice
- Dying
- Death
- Assisted dying
- Bereavement
- Grief
- Life-limiting
- Advanced illness

Included:

- University staff and PhD students listed as members of a palliative and end-of-life care -related research group (excluding those whose primary focus was not palliative and end-of-life care, based on group leaders’ input)
- Researchers with published works or ongoing projects explicitly focused on palliative and end-of-life care
- Researchers who list palliative and end-of-life care as part of their research interests
- Researchers working in palliative and end-of-life care -related areas (e.g., global health, paediatrics, professional education, relevant societies)
- Researchers with Honorary or Emeritus status
- PhD students specifically researching palliative and end-of-life care
- Trial staff involved in palliative and end-of-life care clinical trials (e.g., trial coordinators)
- Researchers without explicitly stated palliative and end-of-life care interests but with recent (past 3years) or multiple (3+) palliative and end-of-life care -related publications
- Researchers in death/dying (e.g., social sciences), only if their work is within health and social care

Excluded:

- Non-research staff (e.g., administrative staff)
- Clinical academics without research involvement
- Undergraduate and master's students
- Visiting academics
- Researchers who do not explicitly state palliative and end-of-life care in their research interests
- Researchers with only one or two palliative and end-of-life care -related publications that are relatively old (e.g., more than 3 years ago)
