# Supplementary material for: Growing palliative and end-of-life care research in the United Kingdom: Findings from a mixed methods survey
Source: PLoS One. 2026 Sep 15;21(9):e0357024. doi: 10.1371/journal.pone.0357024 (PMC13577371; doi:10.1371/journal.pone.0357024)
Supplement: Supplementary file 4 [file pone.0357024.s003.docx]

**S3 Table.** **Characteristics of survey participants.**

**n=256 (%)**

| **Characteristics** | **Number** | **Percentage** |
| --- | --- | --- |
| **Gender** |  |  |
| Female | 205 | 80% |
| Male | 47 | 18% |
| Non-binary/Prefer to self-describe/Prefer not to say | 4 | 2% |
| **Age** |  |  |
| 18-34 | 44 | 17% |
| 35-44 | 60 | 23% |
| 45-54 | 73 | 29% |
| 55-64 | 64 | 25% |
| 65+ | 14 | 6% |
| Prefer not to say | 1 | <1% |
| **Ethnicity** |  |  |
| White | 218 | 85% |
| Asian | 10 | 4% |
| Black | 7 | 3% |
| Mixed | 8 | 3% |
| Prefer not to say/ Other/unknown | 13 | 5% |
| **Area of the UK** |  |  |
| England | 194 | 76% |
| Scotland/Wales/Northern Ireland/Not specified | 62 | 24% |
| **First generation or first in family to attend university** |  |  |
| Yes | 131 | 51% |
| No | 121 | 47% |
| Unsure/Not applicable | 4 | 2% |
| **Disability** |  |  |
| Yes | 37 | 14% |
| No | 214 | 84% |
| Prefer not to say | 5 | 2% |
| **Caring responsibilities** |  |  |
| Yes | 144 | 56% |
| No | 112 | 44% |
| **Roles** |  |  |
| Professor/Group Leader/Director/Head of Department or equivalent | 45 | 18% |
| Associate Professor/Senior Lecturer/Reader or equivalent | 39 | 15% |
| Assistant Professor/Lecturer or equivalent | 23 | 9% |
| Senior Research Fellow/Research Fellow/Senior Research Associate/Research Associate or equivalent | 50 | 20% |
| Clinical Researcher/Clinical Academic/Clinical Research Training Fellow or equivalent | 22 | 9% |
| Research Assistant or equivalent | 11 | 4% |
| PhD Student | 27 | 11% |
| Other* (e.g., Retired; Honorary; Research Technician) | 39 | 15% |
| **Employment status** |  |  |
| Full-time | 172 | 67% |
| Part-time | 65 | 25% |
| Not applicable/Other | 19 | 8% |
| **Employment contract type** |  |  |
| Permanent contract | 120 | 47% |
| Fixed-term contract | 93 | 36% |
| Don't know | 10 | 4% |
| Other | 33 | 13% |
| **Primary research discipline** |  |  |
| Nursing | 59 | 23% |
| Medicine | 44 | 17% |
| Social Science (including Education and Humanities) | 54 | 21% |
| Health Sciences | 27 | 11% |
| Psychology | 24 | 9% |
| Public Health | 14 | 5% |
| Allied Health Profession (including Social Work (n=6)) | 16 | 6% |
| Other | 18 | 7% |
| **Primary employment sector** |  |  |
| University | 201 | 79% |
| Hospice | 23 | 9% |
| Hospital | 14 | 5% |
| Community/Care home/ Not-for-profit research Institute (e.g., charity-funded) | 8 | 3% |
| Other | 10 | 4% |
| **Years involved in palliative and end-of-life care research (including PhD time if relevant)** |  |  |
| Less than 1 year | 12 | 5% |
| 1 year to less than 5 years | 83 | 32% |
| 5 years to less than 10 years | 63 | 25% |
| 10 years to less than 20 years | 67 | 26% |
| 20 years or more | 29 | 11% |
| Prefer not to say/Other | 2 | 1% |
