# Supplementary material for: Growing palliative and end-of-life care research in the United Kingdom: Findings from a mixed methods survey
Source: PLoS One. 2026 Sep 15;21(9):e0357024. doi: 10.1371/journal.pone.0357024 (PMC13577371; doi:10.1371/journal.pone.0357024)
Supplement: Supplementary file 5 [file pone.0357024.s004.docx]

**S4 Figure. Lack of funding as a barrier by research role**

|  |  |
| --- | --- |
| Other (n=39) | 41% |
| PhD Student (n=27) | 30% |
| Research Assistant or equivalent (n=11) | 73% |
| Clinical Academic or equivalent (n=22) | 59% |
| Senior Research Fellow/Research Fellow or equivalent (n=50) | 64% |
| Assistant Professor/Lecturer or equivalent (n=23) | 61% |
| Associate Professor/Senior Lecturer or equivalent (n=39) | 69% |
| Professor or equivalent (n=45) | 56% |
