# Supplementary material for: Growing palliative and end-of-life care research in the United Kingdom: Findings from a mixed methods survey
Source: PLoS One. 2026 Sep 15;21(9):e0357024. doi: 10.1371/journal.pone.0357024 (PMC13577371; doi:10.1371/journal.pone.0357024)
Supplement: Supplementary file 6 [file pone.0357024.s005.docx]

**S5 Figure. Lack of funding as a barrier by discipline** (N, %)

|  |  |
| --- | --- |
| Other (n=18) | 67% |
| Public Health (n=14) | 36% |
| Medicine (n=44) | 45% |
| Allied Health Profession, Social Work (n=16) | 50% |
| Nursing (n=59) | 53% |
| Psychology (n=24) | 54% |
| Health Sciences (n=27) | 59% |
| Social Science, Education, Humanities (n=54) | 70% |
