# Supplementary material for: Growing palliative and end-of-life care research in the United Kingdom: Findings from a mixed methods survey
Source: PLoS One. 2026 Sep 15;21(9):e0357024. doi: 10.1371/journal.pone.0357024 (PMC13577371; doi:10.1371/journal.pone.0357024)
Supplement: Supplementary file 7 [file pone.0357024.s010.docx]

University of Edinburgh Medical School
 Doorway 6, Medical School
 EDINBURGH
 EH8 9AG

United Kingdom

18.08.2026

Dear Dr Singh,

We are very grateful for your comments and those of the reviewers on our paper entitled “*Growing palliative and end-of-life care research in the United Kingdom: Findings from a mixed methods survey*”. We have now reviewed all comments and show how we responded to each point-by-point below. We feel that these amendments have strengthened our manuscript, particularly the description of mixed method design. Please find attached both a tracked-changes version and a clean copy of the revised manuscript.

We confirm that the funder (Marie Curie, UK) had no role in study design, data collection and analysis, decision to publish, or preparation of the manuscript. The funder provided support in the form of a research fellowship for AF *[Grant reference MCRFS-20-101]* and salary for IO *[Grant reference MCCRP-24-01]*, but did not have any additional role in the study. The specific roles of these authors are articulated in the ‘author contributions’ section.

I am on holiday between 29^th^ June and 13^th^ of July, but will be available outside of this period to respond any correspondence.

Thank you again for your interest in our paper, and we look forward to hearing from you in due course.

Yours sincerely,

Anne Finucane

**Response to Reviewers**

*“Growing palliative and end-of-life care research in the United Kingdom: Findings from a mixed methods survey”*

Page and line numbers refer to the clean revised manuscript.

| Reviewer Feedback | Author Response |
| --- | --- |
| **Reviewer 1** | |
| Well done on this quality piece of work. I found it interesting and am sure it will be useful to inform future support for pall care researchers in the UK. I have a few very minor suggestions for your consideration, which may further strengthen the manuscript. | Thank you for your careful review of our manuscript. We greatly appreciate your constructive comments and have revised the manuscript accordingly. |
| - Please check the paper for consistent use of PEOLC (sometimes written in full?). | Thank you for pointing this out. We have defined palliative and end-of-life care (PEOLC) at first mention and used the abbreviation PEOLC consistently throughout the manuscript. |
| - Should the title read 'mixed-methods'? | We would prefer to retain ‘mixed methods’ without a hyphen as this word format aligns with the format used by Creswell and Plano Clark who have written extensively about the mixed methods research. Though we are aware that mixed-methods is also sometimes used. |
| - I wonder if the first sentence in the paper should be referenced? | The existing reference (Sleeman et al 2019) covers both the first and second sentence. We now show this by adding it to the end of the first sentence to enhance clarity. |
| - In paragraph 4 in the background, it might be worth noting where the studies were conducted, to position them with your study (focused on UK). | Thank you, we have clarified where the existing studies were conducted and rewritten the sentence:  *“*Existing **UK-based** studies on researcher perspectives regarding palliative care research activity have been narrow in focus and limited to specific professional groups or regions*” (page 5, Line 80)* |
| - In table 1, I would recommend adding a column for N, next to %. | Thank you for this suggestion. As counts are not available for the Wellcome study, we have retained percentages only in the table to maintain accuracy and consistency. |
| - In table 1, I recommend the heading include what the number is indicating (agreed/strongly agreed)... rather than a footnote. | Thank you for this suggestion. We have revised the column heading to clarify that the percentages indicate agreement. We have retained the footnotes to explain how agreement was defined in each survey, as the response scales differed between our survey and the Wellcome Trust survey. |
| - There is limited focus on differences between your study and the Wellcome Trust Survey. Overall, I thought the results indicated a better culture in pall care research in many areas! I know this isn't the main focus of the study, but I found it interesting. | Thank you for this comment. We have highlighted the comparison more clearly on Page 11, Line 222  *“Overall, participants conducting palliative care research were more positive about all aspects of their working environment compared to the general research workforce as indicated by the Wellcome Trust Survey.”*  We have also added a line in the first paragraph of the discussion to emphasize the more favourable perceptions of research culture amongst those working in palliative car compared to other fields:  *“Indeed, when compared to the perspectives of researchers across a range of fields, those involved in conducting palliative care research were general more positive about nearly all aspects of their working environment.*” (L507-510, p24). |
| **Reviewer 2** | |
| The manuscript addresses a relevant and underexplored topic concerning the sustainability and development of the palliative and end-of-life care (PEOLC) research workforce in the United Kingdom. The study presents potentially important findings regrcher retention, and research capacity building in the field. The manuscript is generally well structured and has scientific merit. However, some methodological and analytical aspects require further clarification and development prior to publication. | Thank you for taking the time to conduct a detailed and thorough review of our manuscript. Your comments are much appreciated, and we have made changes to the manuscript as a result, which we believe strengthen the paper. |
| MAJOR COMMENTS  1. The manuscript states that it employed a convergent mixed methods design (“questionnaire variant of the convergent mixed methods research design”). However, the integration between the quantitative and qualitative components is insufficiently articulated throughout the manuscript. Currently, the qualitative findings mainly serve as illustrations of the quantitative results.  The authors should clarify:  (i) at which stages integration occurred;  (ii) how qualitative findings modified, deepened, or explained the quantitative findings;  (iii) which conclusions were only possible due to the combination of methods. The statement: “Quantitative and qualitative data were then synthesized and consolidated through iterative discussions within the research team” is overly broad for a mixed methods study. Greater methodological detail regarding the integration process is recommended. | Thank you for this helpful comment. We agree that the previous description of data integration was too broad and we now describe the process in more depth in response to the reviewers' helpful comments.  We have added a new section in the Methods entitled Data integration which describes the process in more detail. (Page 9 Line 179-189):  *“Data integration*  *Following collection of both quantitative and qualitative data concurrently, the research team analysed each dataset separately prior to consolidation and merging. During integration, the research team compared the findings from both datasets. This involved comparing common findings across each dataset and considering ways the results confirm, disconfirm and expand upon each other. The integrated results were presented via joint displays in PowerPoint, and then shared, discussed and refined during full team meetings. The qualitative findings did not modify the quantitative results themselves; rather, they deepened and expanded their interpretation. The integration of both datasets allowed development of results and interpretations that expanded our understanding of participant perspectives on conducting PEOLC research in the UK with qualitative findings contextualising and adding detail to the quantitative findings.”* |
| 2. The authors state that the design allowed for “enriching the overall findings,” but this is insufficient as a methodological rationale.  The manuscript would benefit from explicitly clarifying:  (1) why quantitative data alone would have been insufficient;  (2) why qualitative data were necessary;  (3) what the intended purpose of integration was (e.g., complementarity, expansion, triangulation, development). Without this clarification, the study reads more as a survey containing open-ended questions than as a fully integrated mixed methods study. | We have amended the section on study design section to further clarify our rationale for conducted a mixed-method survey and the intended purpose of integration. (Page 6, Line 105-111;, see Study Design).  *“We conducted a convergent mixed-methods study adopting the questionnaire variant of the convergent mixed methods research design [13,14]. We designed an online questionnaire consisting of closed- and open-ended questions; whereby responses from the open-ended questions were used to triangulate and expand upon the quantitative findings. The qualitative data provided further detail on participants’ experiences, priorities and suggestions that could not be captured through the closed-ended survey items alone.”* |
| 3. The “Strengths and limitations” section partially overstates the depth of methodological integration. The authors state: “the integration of quantitative and qualitative data allowed for a rich understanding” However, given that the qualitative data derive from open-ended online survey responses, the interpretive depth is inherently more limited than would be expected from interviews or focus groups. The authors are encouraged to moderate this statement and explicitly acknowledge that: (a) the design enabled contextualization of the quantitative findings; (b) however, the qualitative depth was constrained by the nature of the data collection method. | Thank you for this helpful comment. We have revised the Strengths and Weakness to incorporate the reviewers' insightful suggestion. *(Page 30, Line 652-659)*  *“Another strength was that we employed a mixed-methods design, with qualitative findings enabling contextualisation and expansion of quantitative findings. The integration of quantitative and qualitative data helped contextualise the quantitative findings and provided a more detailed understanding of the factors shaping the PEOLC research environment and workforce. However, the qualitative depth may have been constrained by the data collection method with a reliance on open-ended survey responses rather than in-depth interviews or focus groups. This limitation is typical of the ‘questionnaire variant’ of convergent mixed method designs, where the qualitative component is often an add-on to the quantitative elements.”* |
| 4. The manuscript could better explore divergent findings or tensions across datasets. For example, participants reported collaborative and positive working environments, while simultaneously reporting high levels of job insecurity, workload burden, and likelihood of leaving the field. This contradiction is analytically interesting and deserves deeper exploration in the discussion section:  How can positive work culture coexist with substantial precarity? Is there a strong professional or moral commitment sustaining engagement despite instability? Is academic precarity becoming normalized within the field? At present, these tensions are described but not sufficiently interpreted. | Thank you for highlighting this important point.  We have added a paragraph to the Discussion, to explore how positive working cultures can coexist with substantial precarity. These findings may reflect different levels of the research environment: supportive teams may improve researchers’ day-to-day experiences, while structural issues such as limited funding, job insecurity and unclear career pathways remain unresolved. This interpretation is consistent with our quantitative findings. PEOLC researchers reported more positive views of their research environment than the wider research population. However, only 69% believed that they were likely to continue working in PEOLC research over the next five years, while 90% of respondents wished to do so. Although these findings raise the possibility that precarity has become normalised within the field, our data do not allow us to draw firm conclusions.  *“This coexistence of positive and collaborative working environments with high levels of insecurity is not necessarily contradictory. Nor is the gap between researchers’ strong desire to remain in PEOLC research and their lower expectation of being able to do so. Supportive colleagues and a positive, collaborative team culture may improve researchers’ individual experiences. However, structural problems such as lack of funding, job insecurity, and unclear career pathways persist beyond the control of individual researchers and research teams.*  *To sustain and grow palliative care research, there is a need for action at organizational, community, policy and societal levels. At a broad level this includes greater access to funding for researchers at all career stages; increased visibility of palliative care as a vital research area; greater collaboration and networking opportunities, protected research time and further efforts to enhance research workforce diversity.”*  *(page 24, Line 514 - 524)* |
| 5. The manuscript describes inequalities related to gender, age, and disability, but the discussion remains largely descriptive. The authors are encouraged to further explore:  (a) how precarious employment may disproportionately affect women and researchers with caring responsibilities;  (b) how late academic entry pathways affect access to funding;  (c) how prevailing productivity models in academia may reproduce structural inequities.  This would strengthen the manuscript’s critical contribution. | Thank you for this important comment. We have added some sentences to the Discussion to consider how inequalities may be reproduced through structural features of academic careers.   (a) We now discuss how precarious employment may disproportionately affect researchers with caring responsibilities, including where periods of leave affect employment continuity.   *“Precarious employment may disproportionally affect researchers with caring responsibility by disrupting employment continuity and making it more difficult to remain in research following a period of leave. [Davies et al 2022]” (page 29, Line 623-625)*  *Davies JM, Brighton LJ, Reedy F, et al. Maternity provision, contract status, and likelihood of returning to work: Evidence from research intensive universities in the UK. Gend Work Organ 2022; 29: 1495–1510.*   (b) We also consider how researchers entering academia later or following non-linear career pathways may face difficulties within funding schemes based on conventional early-career trajectories. (c) Finally, we have added discussion of how prevailing productivity models, including expectations of continuous output, success in securing funding, and linear career progression, may disadvantage researchers with caring responsibilities, disabilities or career interruptions, potentially reducing workforce diversity and narrowing the scope of research within the field.   *“Age-related inequalities in access to funding were also identified in the qualitative findings. Researchers entering academia later in their careers may face challenges accessing funding opportunities which are designed around conventional trajectories. Although such researchers may be at an early stage of their academic careers, they may not fit easily within eligibility criteria or expectations that assume linear progression through academic training. Researchers with disabilities may also be disadvantaged by prevailing expectations of continuous output, success in securing funding, and linear career progression. These findings suggest that current research structures may unintentionally disadvantage those whose career pathways or personal circumstances do not align with conventional academic progression. This may reduce workforce diversity and narrow the scope of research topics required to ensure PEOLC research reflects the needs of diverse populations.” (page 29, Line 627-632)* |
| MINOR COMMENTS  6. Figure 4 provides a useful visual summary of factors influencing workforce sustainability. However, the manuscript would benefit from a clearer explanation in the Methods section regarding how the five themes were operationally derived through integration of quantitative and qualitative findings. | Thank you for this comment. We have revised the Methods section to clarify the analytical process.  We have amended part of the data analysis section:  *Within each of these categories, free-text responses were inductively coded, with researchers applying more detailed codes. The research team met weekly to compare codes across all categories and to develop a coding framework. Related codes addressing interconnected connected issues were re-grouped into five overarching themes. The five final themes identified therefore represent cross-cutting issues, drawing on both quantitative and qualitative data, rather than corresponding directly to the four predetermined categories*.  *. (Page 8, Line 170-176))* |
| 7. It would be useful to report more clearly: - how many participants responded to the open-ended questions; - the average length of qualitative responses; - the overall volume of qualitative material analyzed. These details would help readers assess the robustness of the qualitative component. | Thank you for this suggestion. We have added the number of participants who responded to at least one of the six open-ended questions and the total number of open-ended responses analysed. Given that the qualitative data were used to mainly provide context for the quantitative findings, we have not included the average response length.  The following has been added to the results section page 10, Line 198-201:  *“All quantitative questions were compulsory, whereas the six open-ended questions were optional. Of the 256 participants, 244 responded to at least one open-ended question, yielding 966 qualitative responses all together. (excluding non-substantive responses such as "unsure", "N/A")”* |
| Overall recommendation  The study addresses an important issue and offers a potentially valuable contribution to strengthening palliative care research capacity. However, the manuscript requires greater methodological clarity regarding the mixed methods design, particularly in relation to data integration and the inferential contribution generated by combining methods. | Thank you very much for your valuable comments. We have amended the paper in line with your suggestions. |
| **Journal Requirements** | |
| 1.Please ensure that your manuscript meets PLOS ONE's style requirements, including those for file naming. The PLOS ONE style templates can be found at  https://journals.plos.org/plosone/s/file?id=wjVg/PLOSOne_formatting_sample_main_body.pdf  and  [https://journals.plos.org/plosone/s/file?id=ba62/PLOSOne_formatting_sample_title_authors_affiliations.pdf](https://eur02.safelinks.protection.outlook.com/?url=https%3A%2F%2Ftrack.editorialmanager.com%2FCL0%2Fhttps%3A%252F%252Fjournals.plos.org%252Fplosone%252Fs%252Ffile%253Fid%3Dba62%252FPLOSOne_formatting_sample_title_authors_affiliations.pdf%2F1%2F010f019e71e073bd-07635b30-5b3e-45a0-9705-0c1696deae96-000000%2F_XNmx8MDU-rJIB6Cf3qq07QAHUasAbzzT1KMPebn-Kk%3D258&data=05%7C02%7Ciokamoto001%40dundee.ac.uk%7C997a5b0871ac407a185008debd56f6d5%7Cae323139093a4d2a81a65d334bcd9019%7C0%7C0%7C639156378916591938%7CUnknown%7CTWFpbGZsb3d8eyJFbXB0eU1hcGkiOnRydWUsIlYiOiIwLjAuMDAwMCIsIlAiOiJXaW4zMiIsIkFOIjoiTWFpbCIsIldUIjoyfQ%3D%3D%7C0%7C%7C%7C&sdata=FngzEwGjMCFImu9RXlRCl%2FezdKx%2FpA170nHs3oM%2Fxfg%3D&reserved=0) | We have rechecked requirements. We have removed ORCID from our manuscript and renamed files in line with suggestions. We confirm that our manuscript meets PLOS One requirements. |
| 2. Please note that funding information should not appear in any section or other areas of your manuscript. We will only publish funding information present in the Funding Statement section of the online submission form. Please remove any funding-related text from the manuscript. | Information on funding has now been removed. |
| 3. We note that the grant information you provided in the ‘Funding Information’ and ‘Financial Disclosure’ sections do not match.  When you resubmit, please ensure that you provide the correct grant numbers for the awards you received for your study in the ‘Funding Information’ section. | This has now been rectified. We mention both awards in the Current Funding Sources List. |
| 4. Thank you for stating the following financial disclosure:  “This project was funded by Marie Curie [Grant reference MCCRP-24-01].  AF is funded by a Marie Curie Senior Research Fellowship [Grant reference MCRFS-20-101].  FM is a UK National Institute for Health and Care Research (NIHR) Senior Investigator.  The views expressed in this article are those of the author(s) and not necessarily those of Marie Curie, the NIHR, or the UK Department of Health and Social Care.”  Please state what role the funders took in the study.  If the funders had no role, please state: "The funders had no role in study design, data collection and analysis, decision to publish, or preparation of the manuscript."  If this statement is not correct you must amend it as needed.  Please include this amended Role of Funder statement in your cover letter; we will change the online submission form on your behalf. | We confirm that the funders had no role in study design, data collection and analysis, decision to publish, or preparation of the manuscript.  We note this in our cover letter and in our funding statement. |
| 5. We note that you have indicated that there are restrictions to data sharing for this study. PLOS only allows data to be available upon request if there are legal or ethical restrictions on sharing data publicly. For more information on unacceptable data access restrictions, please see http://journals.plos.org/plosone/s/data-availability#loc-unacceptable-data-access-restrictions.  Before we proceed with your manuscript, please address the following prompts:  a) If there are ethical or legal restrictions on sharing a de-identified data set, please explain them in detail (e.g., data contain potentially identifying or sensitive patient information, data are owned by a third-party organization, etc.) and who has imposed them (e.g., a Research Ethics Committee or Institutional Review Board, etc.). Please also provide contact information for a data access committee, ethics committee, or other institutional body to which data requests may be sent.  b) If there are no restrictions, please upload the minimal anonymized data set necessary to replicate your study findings to a stable, public repository and provide us with the relevant URLs, DOIs, or accession numbers. For a list of recommended repositories, please see  https://journals.plos.org/plosone/s/recommended-repositories. You also have the option of uploading the data as Supporting Information files, but we would recommend depositing data directly to a data repository if possible.  We will update your Data Availability statement on your behalf to reflect the information you provide. | We have uploaded the de-identified datasets.  We will share all our data in the Edinburgh University DataShare prior to publication (if accepted). We will provide a DOI to this prior to publication if appropriate. |
| 6. When completing the data availability statement of the submission form, you indicated that you will make your data available on acceptance. We strongly recommend all authors decide on a data sharing plan before acceptance, as the process can be lengthy and hold up publication timelines. Please note that, though access restrictions are acceptable now, your entire data will need to be made freely accessible if your manuscript is accepted for publication. This policy applies to all data except where public deposition would breach compliance with the protocol approved by your research ethics board. If you are unable to adhere to our open data policy, please kindly revise your statement to explain your reasoning and we will seek the editor's input on an exemption. Please be assured that, once you have provided your new statement, the assessment of your exemption will not hold up the peer review process. | We have uploaded the de-identified datasets. |
| 7. Thank you for stating the following financial disclosure:   “This project was funded by Marie Curie [Grant reference MCCRP-24-01].  AF is funded by a Marie Curie Senior Research Fellowship [Grant reference MCRFS-20-101].  FM is a UK National Institute for Health and Care Research (NIHR) Senior Investigator.  The views expressed in this article are those of the author(s) and not necessarily those of Marie Curie, the NIHR, or the UK Department of Health and Social Care.”  We note that one or more of the authors is affiliated with the funding organization, indicating the funder may have had some role in the design, data collection, analysis or preparation of your manuscript for publication; in other words, the funder played an indirect role through the participation of the co-authors. If the funding organization did not play a role in the study design, data collection and analysis, decision to publish, or preparation of the manuscript and only provided financial support in the form of authors' salaries and/or research materials, please do the following:  1. Review your statements relating to the author contributions, and ensure you have specifically and accurately indicated the role(s) that these authors had in your study. These amendments should be made in the online form.  2. Confirm in your cover letter that you agree with the following statement, and we will change the online submission form on your behalf:  “The funder provided support in the form of salaries for authors [insert relevant initials], but did not have any additional role in the study design, data collection and analysis, decision to publish, or preparation of the manuscript. The specific roles of these authors are articulated in the ‘author contributions’ section. | Now amended and recorded in the Financial Disclosure section.  *“This project was funded by Marie Curie [Grant reference MCCRP-24-01]. AF is funded by a Marie Curie Senior Research Fellowship [Grant reference MCRFS-20-101]. The funder provided support in the form of salaries for authors [AF and IO], but did not have any additional role in the study design, data collection and analysis, decision to publish, or preparation of the manuscript. The specific roles of these authors are articulated in the ‘author contributions’ section*  *FM is a UK National Institute for Health and Care Research (NIHR) Senior Investigator. The views expressed in this article are those of the author(s) and not necessarily those of Marie Curie, the NIHR, or the UK Department of Health and Social Care. “* |
| 8. We note that Figure 4 in your submission contain copyrighted images. All PLOS content is published under the Creative Commons Attribution License (CC BY 4.0), which means that the manuscript, images, and Supporting Information files will be freely available online, and any third party is permitted to access, download, copy, distribute, and use these materials in any way, even commercially, with proper attribution. For more information, see our copyright guidelines: <http://journals.plos.org/plosone/s/licenses-and-copyright>.  We require you to either (1) present written permission from the copyright holder to publish these figures specifically under the CC BY 4.0 license, or (2) remove the figures from your submission:   1. You may seek permission from the original copyright holder of Figure 4 to publish the content specifically under the CC BY 4.0 license.   We recommend that you contact the original copyright holder with the Content Permission Form (http://journals.plos.org/plosone/s/file?id=7c09/content-permission-form.pdf) and the following text:  “I request permission for the open-access journal PLOS ONE to publish XXX under the Creative Commons Attribution License (CCAL) CC BY 4.0 (http://creativecommons.org/licenses/by/4.0/). Please be aware that this license allows unrestricted use and distribution, even commercially, by third parties. Please reply and provide explicit written permission to publish XXX under a CC BY license and complete the attached form.”   Please upload the completed Content Permission Form or other proof of granted permissions as an "Other" file with your submission.   In the figure caption of the copyrighted figure, please include the following text: “Reprinted from [ref] under a CC BY license, with permission from [name of publisher], original copyright [original copyright year].”   2. If you are unable to obtain permission from the original copyright holder to publish these figures under the CC BY 4.0 license or if the copyright holder’s requirements are incompatible with the CC BY 4.0 license, please either i) remove the figure or ii) supply a replacement figure that complies with the CC BY 4.0 license. Please check copyright information on all replacement figures and update the figure caption with source information. If applicable, please specify in the figure caption text when a figure is similar but not identical to the original image and is therefore for illustrative purposes only. | Please note that Figure 4 was originally created by the corresponding author (AF) using Napkin AI. Napkin AI is artificial intelligence tool designed to transform text descriptions into diagrams. It combines natural language processing with computer vision to create professional-looking visualizations from simple text inputs. Images created in Napkin are not subject to copyright. For further information please see the Napkin AI site:  [Can I use Napkin AI visuals without copyright issues? \| Napkin AI Help Center](https://help.napkin.ai/en/articles/10027658-can-i-use-napkin-ai-visuals-without-copyright-issues)  We now note the use of Napkin under the image for transparency:  “*Fig 4 was created by the corresponding author (AF) using Napkin AI, an artificial intelligence tool designed to transform text descriptions into diagrams.”* |
| 9. We note that there is identifying data in the Supporting Information file  “S3 Table. Participant characteristics.docx”. Due to the inclusion of these potentially identifying data, we have removed this file from your file inventory. Prior to sharing human research participant data, authors should consult with an ethics committee to ensure data are shared in accordance with participant consent and all applicable local laws.  Data sharing should never compromise participant privacy. It is therefore not appropriate to publicly share personally identifiable data on human research participants. The following are examples of data that should not be shared:  -Name, initials, physical address  -Ages more specific than whole numbers  -Internet protocol (IP) address  -Specific dates (birth dates, death dates, examination dates, etc.)  -Contact information such as phone number or email address  -Location data  -ID numbers that seem specific (long numbers, include initials, titled “Hospital ID”) rather than random (small numbers in numerical order)  Data that are not directly identifying may also be inappropriate to share, as in combination they can become identifying. For example, data collected from a small group of participants, vulnerable populations, or private groups should not be shared if they involve indirect identifiers (such as sex, ethnicity, location, etc.) that may risk the identification of study participants.  Additional guidance on preparing raw data for publication can be found in our Data Policy (https://journals.plos.org/plosone/s/data-availability#loc-human-research-participant-data-and-other-sensitive-data) and in the following article: http://www.bmj.com/content/340/bmj.c181.long.  Please remove or anonymize all personal information (<specific identifying information in file to be removed>), ensure that the data shared are in accordance with participant consent, and re-upload a fully anonymized data set. Please note that spreadsheet columns with personal information must be removed and not hidden as all hidden columns will appear in the published file. | We have revised S3 and can confirm that this file does not contain any data that would compromise participant privacy. We have aggregated any categories consisting of 5 or less responses to ensure anonymity. The file now contains summary data to describe the sample, but it does not contain any data at an individual level, or categories with very small numbers of participants, so it would be impossible to identify individuals from this information. |
| 10. If the reviewer comments include a recommendation to cite specific previously published works, please review and evaluate these publications to determine whether they are relevant and should be cited. There is no requirement to cite these works unless the editor has indicated otherwise.   Additional Editor Comments: Please could you address the reviewer comments below and in particular, clarify the methodology. Abbreviations should also be avoided in the abstract.  [Note: HTML markup is below. Please do not edit.] | We are grateful to the reviewers for their thoughtful comments. We amended our paper in line with suggestions and provide more detail on our mixed methods design.  We have also removed abbreviations from the Abstract, and edited it slightly so it remains within the wordcount limit. |
