# Supplementary material for: Growing palliative and end-of-life care research in the United Kingdom: Findings from a mixed methods survey
Source: PLoS One. 2026 Sep 15;21(9):e0357024. doi: 10.1371/journal.pone.0357024 (PMC13577371; doi:10.1371/journal.pone.0357024)
Supplement: Peer review history

# Peer review: Decision Letter 0

**DOI:** 10.1371/journal.pone.0357024.r001

**Authors:** Gursharan Singh

28 May 2026

Dear Dr. Finucane,

Thank you for submitting your manuscript to PLOS ONE. After careful consideration, we feel that it has merit but does not fully meet PLOS ONE’s publication criteria as it currently stands. Therefore, we invite you to submit a revised version of the manuscript that addresses the points raised during the review process.

Please submit your revised manuscript by Jul 12 2026 11:59PM. If you will need more time than this to complete your revisions, please reply to this message or contact the journal office at plosone@plos.org. When you're ready to submit your revision, log on to https://www.editorialmanager.com/pone/ and select the 'Submissions Needing Revision' folder to locate your manuscript file.

- A letter that responds to each point raised by the academic editor and reviewer(s). You should upload this letter as a separate file labeled 'Response to Reviewers'.
- A marked-up copy of your manuscript that highlights changes made to the original version. You should upload this as a separate file labeled 'Revised Manuscript with Track Changes'.
- An unmarked version of your revised paper without tracked changes. You should upload this as a separate file labeled 'Manuscript'.

If you would like to make changes to your financial disclosure, please include your updated statement in your cover letter. Guidelines for resubmitting your figure files are available below the reviewer comments at the end of this letter.

If applicable, we recommend that you deposit your laboratory protocols in protocols.io to enhance the reproducibility of your results. Protocols.io assigns your protocol its own identifier (DOI) so that it can be cited independently in the future. For instructions see: https://journals.plos.org/plosone/s/submission-guidelines#loc-laboratory-protocols. Additionally, PLOS ONE offers an option for publishing peer-reviewed Lab Protocol articles, which describe protocols hosted on protocols.io. Read more information on sharing protocols at https://plos.org/protocols?utm_medium=editorial-email&utm_source=authorletters&utm_campaign=protocols.

As the corresponding author, your ORCID iD is verified in the submission system and will appear in the published article. PLOS supports the use of ORCID, and we encourage all coauthors to register for an ORCID iD and use it as well. Please encourage your coauthors to verify their ORCID iD within the submission system before final acceptance, as unverified ORCID iDs will not appear in the published article. *Only*  the individual author can complete the verification step; PLOS staff *cannot*  verify ORCID iDs on behalf of authors.

We look forward to receiving your revised manuscript.

Kind regards,

Gursharan K Singh, PhD

Academic Editor

PLOS One

Journal Requirements:

When submitting your revision, we need you to address these additional requirements.

1.Please ensure that your manuscript meets PLOS ONE's style requirements, including those for file naming. The PLOS ONE style templates can be found at

https://journals.plos.org/plosone/s/file?id=wjVg/PLOSOne_formatting_sample_main_body.pdf and

https://journals.plos.org/plosone/s/file?id=ba62/PLOSOne_formatting_sample_title_authors_affiliations.pdf

2. Please note that funding information should not appear in any section or other areas of your manuscript. We will only publish funding information present in the Funding Statement section of the online submission form. Please remove any funding-related text from the manuscript.

3. We note that the grant information you provided in the ‘Funding Information’ and ‘Financial Disclosure’ sections do not match.

When you resubmit, please ensure that you provide the correct grant numbers for the awards you received for your study in the ‘Funding Information’ section.

4. Thank you for stating the following financial disclosure:

“This project was funded by Marie Curie [Grant reference MCCRP-24-01].  AF is funded by a Marie Curie Senior Research Fellowship [Grant reference MCRFS-20-101].  FM is a UK National Institute for Health and Care Research (NIHR) Senior Investigator.

The views expressed in this article are those of the author(s) and not necessarily those of Marie Curie, the NIHR, or the UK Department of Health and Social Care.”

Please state what role the funders took in the study.  If the funders had no role, please state: "The funders had no role in study design, data collection and analysis, decision to publish, or preparation of the manuscript."

If this statement is not correct you must amend it as needed.

Please include this amended Role of Funder statement in your cover letter; we will change the online submission form on your behalf.

5. We note that you have indicated that there are restrictions to data sharing for this study. PLOS only allows data to be available upon request if there are legal or ethical restrictions on sharing data publicly. For more information on unacceptable data access restrictions, please see http://journals.plos.org/plosone/s/data-availability#loc-unacceptable-data-access-restrictions.

Before we proceed with your manuscript, please address the following prompts:

a) If there are ethical or legal restrictions on sharing a de-identified data set, please explain them in detail (e.g., data contain potentially identifying or sensitive patient information, data are owned by a third-party organization, etc.) and who has imposed them (e.g., a Research Ethics Committee or Institutional Review Board, etc.). Please also provide contact information for a data access committee, ethics committee, or other institutional body to which data requests may be sent.

b) If there are no restrictions, please upload the minimal anonymized data set necessary to replicate your study findings to a stable, public repository and provide us with the relevant URLs, DOIs, or accession numbers. For a list of recommended repositories, please see

https://journals.plos.org/plosone/s/recommended-repositories. You also have the option of uploading the data as Supporting Information files, but we would recommend depositing data directly to a data repository if possible.

We will update your Data Availability statement on your behalf to reflect the information you provide.

6. When completing the data availability statement of the submission form, you indicated that you will make your data available on acceptance. We strongly recommend all authors decide on a data sharing plan before acceptance, as the process can be lengthy and hold up publication timelines. Please note that, though access restrictions are acceptable now, your entire data will need to be made freely accessible if your manuscript is accepted for publication. This policy applies to all data except where public deposition would breach compliance with the protocol approved by your research ethics board. If you are unable to adhere to our open data policy, please kindly revise your statement to explain your reasoning and we will seek the editor's input on an exemption. Please be assured that, once you have provided your new statement, the assessment of your exemption will not hold up the peer review process.

7. Thank you for stating the following financial disclosure:

“This project was funded by Marie Curie [Grant reference MCCRP-24-01].  AF is funded by a Marie Curie Senior Research Fellowship [Grant reference MCRFS-20-101].  FM is a UK National Institute for Health and Care Research (NIHR) Senior Investigator.

The views expressed in this article are those of the author(s) and not necessarily those of Marie Curie, the NIHR, or the UK Department of Health and Social Care.”

We note that one or more of the authors is affiliated with the funding organization, indicating the funder may have had some role in the design, data collection, analysis or preparation of your manuscript for publication; in other words, the funder played an indirect role through the participation of the co-authors. If the funding organization did not play a role in the study design, data collection and analysis, decision to publish, or preparation of the manuscript and only provided financial support in the form of authors' salaries and/or research materials, please do the following:

1. Review your statements relating to the author contributions, and ensure you have specifically and accurately indicated the role(s) that these authors had in your study. These amendments should be made in the online form.

2. Confirm in your cover letter that you agree with the following statement, and we will change the online submission form on your behalf:

“The funder provided support in the form of salaries for authors [insert relevant initials], but did not have any additional role in the study design, data collection and analysis, decision to publish, or preparation of the manuscript. The specific roles of these authors are articulated in the ‘author contributions’ section.

8. We note that Figure 4 in your submission contain copyrighted images. All PLOS content is published under the Creative Commons Attribution License (CC BY 4.0), which means that the manuscript, images, and Supporting Information files will be freely available online, and any third party is permitted to access, download, copy, distribute, and use these materials in any way, even commercially, with proper attribution. For more information, see our copyright guidelines: http://journals.plos.org/plosone/s/licenses-and-copyright.

We require you to either (1) present written permission from the copyright holder to publish these figures specifically under the CC BY 4.0 license, or (2) remove the figures from your submission:

1. You may seek permission from the original copyright holder of Figure 4 to publish the content specifically under the CC BY 4.0 license.

We recommend that you contact the original copyright holder with the Content Permission Form (http://journals.plos.org/plosone/s/file?id=7c09/content-permission-form.pdf) and the following text:

“I request permission for the open-access journal PLOS ONE to publish XXX under the Creative Commons Attribution License (CCAL) CC BY 4.0 (http://creativecommons.org/licenses/by/4.0/). Please be aware that this license allows unrestricted use and distribution, even commercially, by third parties. Please reply and provide explicit written permission to publish XXX under a CC BY license and complete the attached form.”

Please upload the completed Content Permission Form or other proof of granted permissions as an "Other" file with your submission.

In the figure caption of the copyrighted figure, please include the following text: “Reprinted from [ref] under a CC BY license, with permission from [name of publisher], original copyright [original copyright year].”

2. If you are unable to obtain permission from the original copyright holder to publish these figures under the CC BY 4.0 license or if the copyright holder’s requirements are incompatible with the CC BY 4.0 license, please either i) remove the figure or ii) supply a replacement figure that complies with the CC BY 4.0 license. Please check copyright information on all replacement figures and update the figure caption with source information. If applicable, please specify in the figure caption text when a figure is similar but not identical to the original image and is therefore for illustrative purposes only.

9. We note that there is identifying data in the Supporting Information file  “S3 Table. Participant characteristics.docx”. Due to the inclusion of these potentially identifying data, we have removed this file from your file inventory. Prior to sharing human research participant data, authors should consult with an ethics committee to ensure data are shared in accordance with participant consent and all applicable local laws.

Data sharing should never compromise participant privacy. It is therefore not appropriate to publicly share personally identifiable data on human research participants. The following are examples of data that should not be shared:

-Name, initials, physical address

-Ages more specific than whole numbers

-Internet protocol (IP) address

-Specific dates (birth dates, death dates, examination dates, etc.)

-Contact information such as phone number or email address

-Location data

-ID numbers that seem specific (long numbers, include initials, titled “Hospital ID”) rather than random (small numbers in numerical order)

Data that are not directly identifying may also be inappropriate to share, as in combination they can become identifying. For example, data collected from a small group of participants, vulnerable populations, or private groups should not be shared if they involve indirect identifiers (such as sex, ethnicity, location, etc.) that may risk the identification of study participants.

Additional guidance on preparing raw data for publication can be found in our Data Policy (https://journals.plos.org/plosone/s/data-availability#loc-human-research-participant-data-and-other-sensitive-data) and in the following article: http://www.bmj.com/content/340/bmj.c181.long.

Please remove or anonymize all personal information (<specific identifying information in file to be removed>), ensure that the data shared are in accordance with participant consent, and re-upload a fully anonymized data set. Please note that spreadsheet columns with personal information must be removed and not hidden as all hidden columns will appear in the published file.

10. If the reviewer comments include a recommendation to cite specific previously published works, please review and evaluate these publications to determine whether they are relevant and should be cited. There is no requirement to cite these works unless the editor has indicated otherwise.

Additional Editor Comments:

Please could you address the reviewer comments below and in particular, clarify the methodology. Abbreviations should also be avoided in the abstract.

[Note: HTML markup is below. Please do not edit.]

Reviewers' comments:

Reviewer's Responses to Questions

**Comments to the Author**

1. Is the manuscript technically sound, and do the data support the conclusions?

Reviewer #1: Yes

Reviewer #2: Yes

**********

2. Has the statistical analysis been performed appropriately and rigorously?

Reviewer #1: Yes

Reviewer #2: N/A

**********

3. Have the authors made all data underlying the findings in their manuscript fully available?

The PLOS Data policy

Reviewer #1: Yes

Reviewer #2: No

**********

4. Is the manuscript presented in an intelligible fashion and written in standard English?

Reviewer #1: Yes

Reviewer #2: Yes

**********

Reviewer #1: Well done on this quality piece of work. I found it interesting and am sure it will be useful to inform future support for pall care researchers in the UK. I have a few very minor suggestions for your consideration, which may further strengthen the manuscript.

- Please check the paper for consistent use of PEOLC (sometimes written in full?).

- Should the title read 'mixed-methods'?

- I wonder if the first sentence in the paper should be referenced?

- In paragraph 4 in the background, it might be worth noting where the studies were conducted, to position them with your study (focused on UK).

- In table 1, I would recommend adding a column for N, next to %.

- In table 1, I recommend the heading include what the number is indicating (agreed/strongly agreed)... rather than a footnote.

- There is limited focus on differences between your study and the Wellcome Trust Survey. Overall, I thought the results indicated a better culture in pall care research in many areas! I know this isn't the main focus of the study, but I found it interesting.

All the best.

Reviewer #2: The manuscript addresses a relevant and underexplored topic concerning the sustainability and development of the palliative and end-of-life care (PEOLC) research workforce in the United Kingdom. The study presents potentially important findings regarding funding, researcher retention, and research capacity building in the field. The manuscript is generally well structured and has scientific merit. However, some methodological and analytical aspects require further clarification and development prior to publication.

MAJOR COMMENTS

1. The manuscript states that it employed a convergent mixed methods design (“questionnaire variant of the convergent mixed methods research design”). However, the integration between the quantitative and qualitative components is insufficiently articulated throughout the manuscript. Currently, the qualitative findings mainly serve as illustrations of the quantitative results. The authors should clarify: (i) at which stages integration occurred; (ii) how qualitative findings modified, deepened, or explained the quantitative findings; (iii) which conclusions were only possible due to the combination of methods. The statement: “Quantitative and qualitative data were then synthesized and consolidated through iterative discussions within the research team” is overly broad for a mixed methods study. Greater methodological detail regarding the integration process is recommended.

2. The authors state that the design allowed for “enriching the overall findings,” but this is insufficient as a methodological rationale. The manuscript would benefit from explicitly clarifying: (1) why quantitative data alone would have been insufficient; (2) why qualitative data were necessary; (3) what the intended purpose of integration was (e.g., complementarity, expansion, triangulation, development). Without this clarification, the study reads more as a survey containing open-ended questions than as a fully integrated mixed methods study.

3. The “Strengths and limitations” section partially overstates the depth of methodological integration. The authors state: “the integration of quantitative and qualitative data allowed for a rich understanding” However, given that the qualitative data derive from open-ended online survey responses, the interpretive depth is inherently more limited than would be expected from interviews or focus groups. The authors are encouraged to moderate this statement and explicitly acknowledge that: (a) the design enabled contextualization of the quantitative findings; (b) however, the qualitative depth was constrained by the nature of the data collection method.

4. The manuscript could better explore divergent findings or tensions across datasets. For example, participants reported collaborative and positive working environments, while simultaneously reporting high levels of job insecurity, workload burden, and likelihood of leaving the field. This contradiction is analytically interesting and deserves deeper exploration in the discussion section: How can positive work culture coexist with substantial precarity? Is there a strong professional or moral commitment sustaining engagement despite instability? Is academic precarity becoming normalized within the field? At present, these tensions are described but not sufficiently interpreted.

5. The manuscript describes inequalities related to gender, age, and disability, but the discussion remains largely descriptive. The authors are encouraged to further explore: (a) how precarious employment may disproportionately affect women and researchers with caring responsibilities; (b) how late academic entry pathways affect access to funding; (c) how prevailing productivity models in academia may reproduce structural inequities. This would strengthen the manuscript’s critical contribution.

MINOR COMMENTS

6. Figure 4 provides a useful visual summary of factors influencing workforce sustainability. However, the manuscript would benefit from a clearer explanation in the Methods section regarding how the five themes were operationally derived through integration of quantitative and qualitative findings.

7. It would be useful to report more clearly:

- how many participants responded to the open-ended questions;

- the average length of qualitative responses;

- the overall volume of qualitative material analyzed.

These details would help readers assess the robustness of the qualitative component.

Overall recommendation

The study addresses an important issue and offers a potentially valuable contribution to strengthening palliative care research capacity. However, the manuscript requires greater methodological clarity regarding the mixed methods design, particularly in relation to data integration and the inferential contribution generated by combining methods.

**********

what does this mean?). If published, this will include your full peer review and any attached files.

If you choose “no”, your identity will remain anonymous but your review may still be made public.

**Do you want your identity to be public for this peer review?**  For information about this choice, including consent withdrawal, please see our Privacy Policy

Reviewer #1: No

Reviewer #2: **Yes:**  Liana Amorim Corrêa Trotte

**********

[NOTE: If reviewer comments were submitted as an attachment file, they will be attached to this email and accessible via the submission site. Please log into your account, locate the manuscript record, and check for the action link "View Attachments". If this link does not appear, there are no attachment files.]

To ensure your figures meet our technical requirements, please review our figure guidelines: https://journals.plos.org/plosone/s/figures

You may also use PLOS’s free figure tool, NAAS, to help you prepare publication quality figures: https://journals.plos.org/plosone/s/figures#loc-tools-for-figure-preparation.

NAAS will assess whether your figures meet our technical requirements by comparing each figure against our figure specifications.

Attachment Submitted filename: Reviwer_Comments.pdf

# Author comment: Author response to Decision Letter 1

**DOI:** 10.1371/journal.pone.0357024.r002

10 Jul 2026

Dear Dr Singh,

We are very grateful for your comments and those of the reviewers on our paper entitled “Growing palliative and end-of-life care research in the United Kingdom: Findings from a mixed methods survey”. We have now reviewed all comments and show how we responded to each point-by-point in the attached file "Response to reviewers_18.06.2026". We believe that these amendments have strengthened our manuscript, particularly the description of mixed method design.

We confirm that the funder (Marie Curie, UK) had no role in study design, data collection and analysis, decision to publish, or preparation of the manuscript. The funder provided support in the form of a research fellowship for AF [Grant reference MCRFS-20-101] and salary for IO [Grant reference MCCRP-24-01], but did not have any additional role in the study. The specific roles of these authors are articulated in the ‘author contributions’ section.

I am on holiday between 29th June and 13th of July, but will be available outside of this period to respond any correspondence.

Thank you again for your interest in our paper, and we look forward to hearing from you in due course.

Yours sincerely,

Anne Finucane and Ikumi Okamoto

Attachment Submitted filename: Response to Reviewers_18.06.2026.docx

# Peer review: Decision Letter 1

**DOI:** 10.1371/journal.pone.0357024.r003

**Authors:** Palash Banik

11 Aug 2026

Growing palliative and end-of-life care research in the United Kingdom:  Findings from a mixed methods survey

PONE-D-26-12640R1

Dear Dr. Finucane,

We’re pleased to inform you that your manuscript has been judged scientifically suitable for publication and will be formally accepted for publication once it meets all outstanding technical requirements.

Within one week, you’ll receive an email detailing the required amendments. When these have been addressed, you’ll receive a formal acceptance letter, and your manuscript will be scheduled for publication.

An invoice will be generated when your article is formally accepted. Please note that if your institution has a publishing partnership with PLOS and your article meets the relevant criteria, all or part of your publication costs will be covered. Please make sure your user information is up-to-date by logging into Editorial Manager at Editorial Manager® and clicking the ‘Update My Information' link at the top of the page. For questions related to billing, please contact billing support.

If your institution or institutions have a press office, please notify them about your upcoming paper to help maximize its impact. If they’ll be preparing press materials, please inform our press team as soon as possible -- no later than 48 hours after receiving the formal acceptance. Your manuscript will remain under strict press embargo until 2 pm Eastern Time on the date of publication. For more information, please contact onepress@plos.org.

Kind regards,

Palash Chandra Banik, MPhil

Academic Editor

PLOS One

Additional Editor Comments (optional):

Reviewers' comments:

Reviewer's Responses to Questions

**Comments to the Author**

Reviewer #1: All comments have been addressed

Reviewer #2: (No Response)

**********

2. Is the manuscript technically sound, and do the data support the conclusions?

Reviewer #1: Yes

Reviewer #2: Yes

**********

3. Has the statistical analysis been performed appropriately and rigorously?

Reviewer #1: Yes

Reviewer #2: Yes

**********

4. Have the authors made all data underlying the findings in their manuscript fully available?

The PLOS Data policy

Reviewer #1: Yes

Reviewer #2: Yes

**********

5. Is the manuscript presented in an intelligible fashion and written in standard English?

Reviewer #1: Yes

Reviewer #2: Yes

**********

Reviewer #1: All comments have been addressed carefully. The manuscript reads well and makes and important contribution.

Reviewer #2: (No Response)

**********

what does this mean?). If published, this will include your full peer review and any attached files.

If you choose “no”, your identity will remain anonymous but your review may still be made public.

**Do you want your identity to be public for this peer review?**  For information about this choice, including consent withdrawal, please see our Privacy Policy

Reviewer #1: No

Reviewer #2: **Yes:**  Liana Amorim Corrêa Trotte

**********

# Editor report: Acceptance letter

**DOI:** 10.1371/journal.pone.0357024.r004

**Authors:** Palash Banik

PONE-D-26-12640R1

PLOS One

Dear Dr. Finucane,

I'm pleased to inform you that your manuscript has been deemed suitable for publication in PLOS One. Congratulations! Your manuscript is now being handed over to our production team.

At this stage, our production department will prepare your paper for publication. This includes ensuring the following:

* All references, tables, and figures are properly cited

* All relevant supporting information is included in the manuscript submission,

* There are no issues that prevent the paper from being properly typeset

You will receive further instructions from the production team, including instructions on how to review your proof when it is ready. Please keep in mind that we are working through a large volume of accepted articles, so please give us a few days to review your paper and let you know the next and final steps.

Lastly, if your institution or institutions have a press office, please let them know about your upcoming paper now to help maximize its impact. If they'll be preparing press materials, please inform our press team within the next 48 hours. Your manuscript will remain under strict press embargo until 2 pm Eastern Time on the date of publication. For more information, please contact onepress@plos.org.

You will receive an invoice from PLOS for your publication fee after your manuscript has reached the completed accept phase. If you receive an email requesting payment before acceptance or for any other service, this may be a phishing scheme. Learn how to identify phishing emails and protect your accounts at https://explore.plos.org/phishing.

If we can help with anything else, please email us at customercare@plos.org.

Thank you for submitting your work to PLOS One and supporting open access.

Kind regards,

PLOS One Editorial Office Staff

on behalf of

Dr. Palash Chandra Banik

Academic Editor

PLOS One
